# Identification of Key Genes Related to Postnatal Testicular Development Based on Transcriptomic Data of Testis in Hu Sheep

**DOI:** 10.3389/fgene.2021.773695

**Published:** 2022-01-25

**Authors:** Haiyue Xu, Wu Sun, Shengwei Pei, Wanhong Li, Fadi Li, Xiangpeng Yue

**Affiliations:** State Key Laboratory of Grassland Agro-ecosystems, Key Laboratory of Grassland Livestock Industry Innovation, Ministry of Agriculture and Rural Affairs, College of Pastoral Agriculture Science and Technology, Lanzhou University, Lanzhou, China

**Keywords:** sheep, testis, transcriptome, DEGs, male reproduction

## Abstract

The selection of testis size can improve the reproductive capacity of livestock used for artificial insemination and has been considered as an important strategy for accelerating the breeding process. Although much work has been done to investigate the mechanisms of testis development in various species, there is little information available in regard to the differences in transcriptomic profiling of sheep testes at different developmental stages. In this work, we aimed to identify differentially expressed genes (DEGs) by RNA-Seq in sheep during different growth stages, including 0 month old (infant, M0), 3 months old (puberty, M3), 6 months old (sexual maturity, M6) and 12 months old (body maturity, M12). A total of 4,606 (2,381 up and 2,225 down), 7,500 (4,368 up and 3,132 down), 15 (8 up and seven down) DEGs were identified in M3_vs_M0, M6_vs_M3, and M12_vs_M6 comparison, respectively. Of which, a number of genes were continuously up-regulated and down-regulated with testicular development, including *ODF3*, *ZPBP1*, *PKDREJ*, *MYBL1*, *PDGFA*, *IGF1*, *LH*, *INSL3*, *VIM*, *AMH*, *INHBA*, *COL1A1*, *COL1A2*, and *INHA*. GO analysis illustrated that DEGs were mainly involved in testis development and spermatogenesis. KEGG analysis identified several important pathways and verified several reproduction-associated DEGs such as *COL1A1*, *COL1A2*, *PDGFA*, and *IGF1.* In addition, two gene modules highly associated with testis development and core genes with testis size were identified using weighted gene co-expression network analysis (WGCNA), including hub genes positively associated with testis size such as *RANBP9*, *DNAH17*, *SPATA4*, *CIB4* and *SPEM1*, and those negatively associated with testis size such as *CD81*, *CSK*, *PDGFA*, *VIM,* and *INHBA*. This study comprehensively identified key genes related to sheep testicular development, which may provide potential insights for understanding male fertility and better guide in animal breeding.

## Introduction

The testis is a specific reproductive organ for male mammals to maintain male characteristics, produce sperm, and secrete androgenic hormones. Because of its high heritability and easy measurement, testis size was widely recognized as one of the important indicates to predicate male reproductive capability as early as the 1970s (Coulter and Foote, 1979). A large number of previous studies based on field data have found that testicular size had significantly positive correlations with ejaculation volume, sperm concentration, and sperm motility, and negative associations with abnormal sperm percentage in cattle, sheep, goat, pig, *etc* (Condorelli et al., 2013; Jacyno et al., 2015; Almaguer et al., 2017; Hagiya et al., 2018). In addition, testicular size not only impacts male reproduction performance but also the age of puberty and the postpartum interval of their genetically related females (Melis et al., 2010; Kastelic, 2014). Therefore, the selection of elite sires based on testicular size is considered as one of the fastest and most effective methods of predicting reproductive capability.

However, the development of mammalian testis is a highly complicated and sophisticated process. This process is controlled by many factors and fundamentally orchestrated through the expression of thousands of protein-encoding genes, which are developmentally regulated during spermatogenesis and play pivotal roles during specific phases of germ cell development. In mouse, a total of 18,837 genes were identified in testis, and they showed distinctly differential expression patterns between infant testis and juvenile, adult testis (Gong et al., 2013). A large number of differentially expressed genes (DEGs) were found to be functional in spermatogenesis and testicular development (Gong et al., 2013). Several studies have been conducted to investigate transcriptomic profiles of pig testes at different developmental stages, and thousands of DEGs were identified between the sexually immature and sexually mature stages. Moreover, some highly DEGs, including *SOX9*, *GATA4*, *FOG2*, *SRY*, *TGF-β*, *PIWIL*, *SPATA,* and *KIT*, were found to play important roles in testicular development and spermatogenesis (Ran et al., 2015; Song et al., 2015; Ran et al., 2017). In addition, the study on transcriptomic profiles of two porcine breeds during different testis development stages revealed genetic similarities and diversities of porcine testicular development, while the detailed molecular mechanisms underlying the gene similarities and diversities of spermatogenesis and testicular development of different pig breeds at different sexual mature ages require further studies (Li et al., 2016). In cattle, Chang et al. identified 1,274 transcripts on the male-specific region of the bovine Y chromosome in testis, indicating that the Y chromosome has high transcriptomic activity in testis development (Chang et al., 2013). A comparison of testicular transcriptomic profiles between fertile yak and infertile cattle-yak revealed that a large number of DEGs are associated with the male infertility of cattle-yak (Cai et al., 2017).

In sheep, very limited studies were conducted to study the differentially transcriptomic profiles of testis during testicular development, while several studies have investigated the response of mRNA expression to nutritional treatment in adult testes. Guan et al. analyzed differentially expressed mRNA in testis between adult sheep that were well-fed and underfed, revealing that DEGs and alternative splicing events are mainly involved in spermatogenesis and germ cell apoptosis in testis (Guan et al., 2017). Another study found that the dosage of dietary vitamin E supplementation had effects on transcriptional activity of antioxidant enzyme-related genes in testis, including *GSTA1*, *GPX3*, *FASLG*, *NPC1L1*, *TDC7,* and *CD36*, which were primarily responsible for the improved reproductive performance promoted by dietary vitamin E (Xu et al., 2016). However, these studies which were confined to a certain period of testicular development were unable to comprehensively unveil the genes related to testicular development, especially DEGs among infancy, pubertal, and sexually mature stages. Thus it was necessary to study the expression profile of sheep testis transcriptome at key developmental stages.

In this study, a well-known indigenous sheep breed, Hu sheep, was selected, which is one of the outstanding and one of the most widely distributed sheep breeds in China. Hu sheep usually reach puberty at 3 months old, and sexual maturity at 6 months old. After reaching 8 months old, Hu rams can usually have their first mating, and they achieve body maturity at 12 months of age (Yue, 1996). Therefore, the current study employed solexa deep sequencing to investigate transcriptomic profiles of ovine testis at infancy, puberty, sexual maturity, and body maturity stages, aiming to unveil candidate genes and regulatory networks related to sheep testicular development.

## Materials and Methods

### Ethics Statement

All experiment protocols were reviewed and approved by the Ethics Committee of the College of Pastoral Agriculture Science and Technology, Lanzhou University (Ethic approval No: 2010-1 and 2010-2). All efforts were taken to minimize animal suffering.

### Samples Collection

Three of each age of healthy Hu rams with similar body weight, from the same farm, were slaughtered at birth (infant, M0), 3 months (puberty, M3), 6 months (sexual maturity, M6), and 12 months (body maturity, M12) to obtain testes and epididymis (Jinchang Zhongtian Sheep Industry Co. Ltd., Gansu, China). The body weights, testis weights, and epididymis weights were measured for rams at M0 (body weight: 3.94 ± 0.42 kg, testis mass: 7.01 ± 0.93 g, epididymis mass: 1.44 ± 0.05 g), M3 (body weight: 14.75 ± 0.24 kg, testis mass: 17.82 ± 3.29 g, epididymis mass: 4.89 ± 1.13 g), M6 (body weight: 41.50 ± 0.03 kg, testis mass: 220.86 ± 1.50 g, epididymis mass: 43.77 ± 6.02 g), and M12 (body weight: 41.10 ± 4.88 kg, testis mass: 319.98 ± 23.49 g, epididymis mass: 50.65 ± 8.14 g), respectively. After immediate dissection on an ice-cold surface, about one cm^3^ tissue of top, middle, and bottom parts of the right testes was mixed and snap-frozen in liquid nitrogen, and then stored at −80°C until RNA extraction.

### RNA Extraction, Library Construction, and Sequencing

Total RNA of each sample was extracted from the testis according to the instruction manual of the TRlzol Reagent (Life Technologies, California, United States). RNA integrity and concentration were checked using an Agilent 2,100 Bioanalyzer (Agilent Technologies, Inc., Santa Clara, CA, United States). The mRNA was isolated by NEBNext Poly (A) mRNA Magnetic Isolation Module (NEB, E7490). The cDNA library was constructed following the manufacturer’s instructions of NEBNext Ultra RNA Library Prep Kit for Illumina (NEB, E7530) and NEBNext Multiplex Oligos for Illumina (NEB, E7500). In brief, the enriched mRNA was fragmented into approximately 200 nt RNA inserts, which were used to synthesize the first-strand cDNA and the second-strand cDNA. The double-stranded cDNA was performed end-repair/dA-tail and adaptor ligation. The suitable fragments were isolated by Agencourt AMPure XP beads (Beckman Coulter, Inc.), and enriched by PCR amplification. In total, twelve cDNA libraries were constructed and named M0R1, M0R2, M0R3, M3R1, M3R2, M3R3, M6R1, M6R2, M6R3, M12R1, M12R2, and M12R3, respectively. Finally, the constructed cDNA libraries of the ovine testis were sequenced on an Illumina HiSeq™ Xten sequencing platform.

### Transcriptome Analysis Using Reference Genome-Based Reads Mapping

The sequenced raw data were filtered using fastp 0.21.0 (Chen et al., 2018) to generate the clean reads to remove the adapter, ambiguous reads (reads with unknown nucleotides “N” more than 5), and low-quality sequences (phred quality < Q20 and unqualified percent >20%) from raw data. The clean reads that were filtered from the raw reads were mapped to the *Ovis aries* reference genome (Oar3.1) using Hisat2 software (Kim et al., 2019). And then HTSeq v0.6.1 (Anders et al., 2015) was used to count the read numbers of every mapped gene. Gene expression levels were normalized by FPKM (fragments per kilobase of exon per million fragments mapped) values based on sequencing depth and gene length.

### Identification of Differential Gene Expression and Annotation

DESeq2 (Anders and Huber, 2010) was employed to evaluate differential gene expression between M3 and M0, M6 and M3, and M12 and M6. The false discovery rate (FDR) control method was used to identify the threshold of the *p*-value in multiple tests in order to compute the significance of the differences. Here, only genes with an absolute value of log2 FC ratio ≥1 and adjusted *p*-value <0.01 were considered DEGs. Finally, genes were compared against various protein databases by BLASTX, including the National Center for Biotechnology Information (NCBI) non-redundant protein (Nr) database, Swiss-Prot database with a cut-off E-value of 10^−5^. Furthermore, genes were searched against the NCBI non-redundant nucleotide sequence (Nt) database using BLASTn by a cut-off E-value of 10^−5^. Genes were retrieved based on the best BLAST hit (highest score) along with their protein functional annotation. In addition, based on the differential expression of the three comparison analysis groups, we further explored key genes by drawing Venn diagrams. Meanwhile, all expression genes of sheep testes were classified based on time development point by k-means R package.

### GO and KEGG Enrichment Analysis

In order to provide more clues to understand the functions of DEGs in sheep testicular development and spermatogenesis, Gene Ontology (GO) enrichment analysis of DEGs from the comparisons between different months of age (M3 vs M0, M6 vs M3, and M12 vs M6) were implemented by Blast2GO program and TopGo (R package) (Conesa et al., 2005). Firstly, the Nr BLAST results were imported into the Blast2GO program. GO annotations for the genes were obtained by Blast2GO. This analysis mapped all of the annotated genes to GO terms in the database and counted the number of genes associated with each term. Perl script was then used to plot GO functional classification for the unigenes with a GO term hit to view the distribution of gene functions. The obtained annotation was enriched and refined using TopGo (R package). KEGG pathways were assigned to the assembled sequences by the perl script. In addition, the key genes that tended to increase and tended to decrease with month age were also used for GO and KEGG analysis in order to better study testicular development and spermatogenesis. Benjamini corrected *p* < 0.05 was considered significantly enriched.

### Validation of mRNA Expression Using qPCR

From a total of six transcripts, three transcripts showing continuous up-regulation with age and three transcripts showing continuous down-regulation with age, were selected to validate the expression level at different development stages in sheep testis tissues. The quantitative PCR (qPCR) was carried out in a 25 μl volume that contained 12.5 μl of SYBR Real-time Green PCR Master Mix (TransGen Biotech, Beijing, China), 0.5 μl of each primer (10 uM), 1 μl of 50 ng/μl cDNA, and 10.5 μl double distilled water. Primers of each protein-coding gene were designed using Primer Premier 5.0 (PREMIER Biosoft International, Palo Alto, CA) and synthesized by Sangon Biological Engineering Technology and Service Co. Ltd. (Shanghai, P. R. China) (Supplementary Table S1). PCR procedure consisted of one cycle at 94°C for 5 min, followed by 40 cycles at 94°C for 40 s, annealing for 30 s, and 72°C for 30 s. The relative expression level was determined using the 2^−ΔΔCt^ method with *β*-actin as internal control (Livak and Schmittgen, 2001). Three independent biological replicates were used for each stage group. Differences were considered significant when *p* < 0.05 from the one-way ANOVA test using SPSS 16.0 (SPSS Inc., Chicago, IL, United States).

### Relationship Between Gene Modules With Sheep Phenotypic Traits

The weighted gene co-expression network analysis (WGCNA) has allowed us to construct a co-expression network for all samples by the WGCNA package in R software (4.1.0) (Langfelder and Horvath, 2008). As recommended by the WGCNA, we used DESeq2 to normalize the matrix of read counts and input the result into WGCNA (Yan et al., 2020). A matrix of pairwise correlations between all pairs of genes across all samples was constructed. An adjacency matrix was then calculated, using the correlation matrix of the expression sets, and transformed into a topological overlap matrix that was then used to derive a distance matrix of hierarchical clustering. Finally, by using the dynamic hybrid cutting method, the genes with similar expression patterns (r > 0.85) were clustered into 16 distinct modules. The correlations were calculated for the relationships between module eigengenes month-old (M0, M3, M6, M12) and phenotypes (testis weight, testis volume, epididymis weight), which here we can find the target module of interests (Cor >0.75, *p* < 0.05). For the target module, we built the regulatory network using the genes inside the module using Cytoscape software (Shannon et al., 2003), from which we can find some of the core genes (Hub genes).

## Results

### Overview of the Sequenced RNAs From Sheep Testes

**FIGURE 1:**
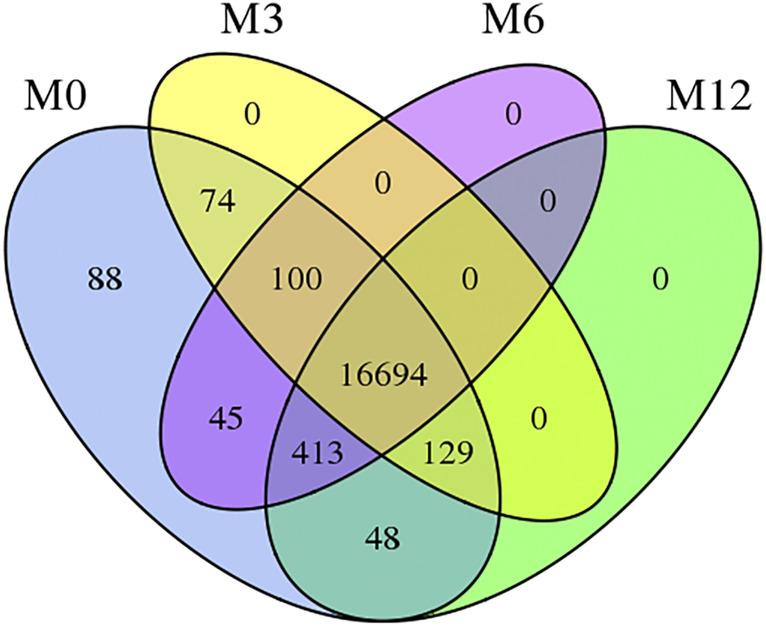
Venn diagram of four development stages expressed genes Note: M0 represents 0-month-old, M3 represents 3-month-old, M6 represents 6-month-old, M12 represents 12-month-old.

The total length of sequence reads generated was approximately 90.959 Gb, of which 20.603, 20.028, 21.411, and 28.916 Gb of clean sequence reads were generated from M0, M3, M6, and M12, respectively (Supplementary Table S2). GC contents of the samples were between 52.15 and 57.15%, and ≥Q30 (%) were 86.88–89.87% (Supplementary Table S3), indicating that RNA-Seq generated a reasonable amount of data for subsequent analysis. Approximately 58.72–75.13% clean reads were mapped to the sheep reference genome (OAR3.1) (Supplementary Table S4); among these, 55.67–73.40% showed unique matches, and 1.62–3.12% showed multiple matches. Furthermore, 17591, 16997, 17252, and 17284 genes were expressed in M0, M3, M6, and M12, respectively. There are 16,694 genes across all the developmental stages and 88, 0, 0, and 0 genes unique to M0, M3, M6, and M12 respectively (Figure 1).

### Identification and Validation of DEGs at Different Testicular Developmental Stages

**FIGURE 2:**
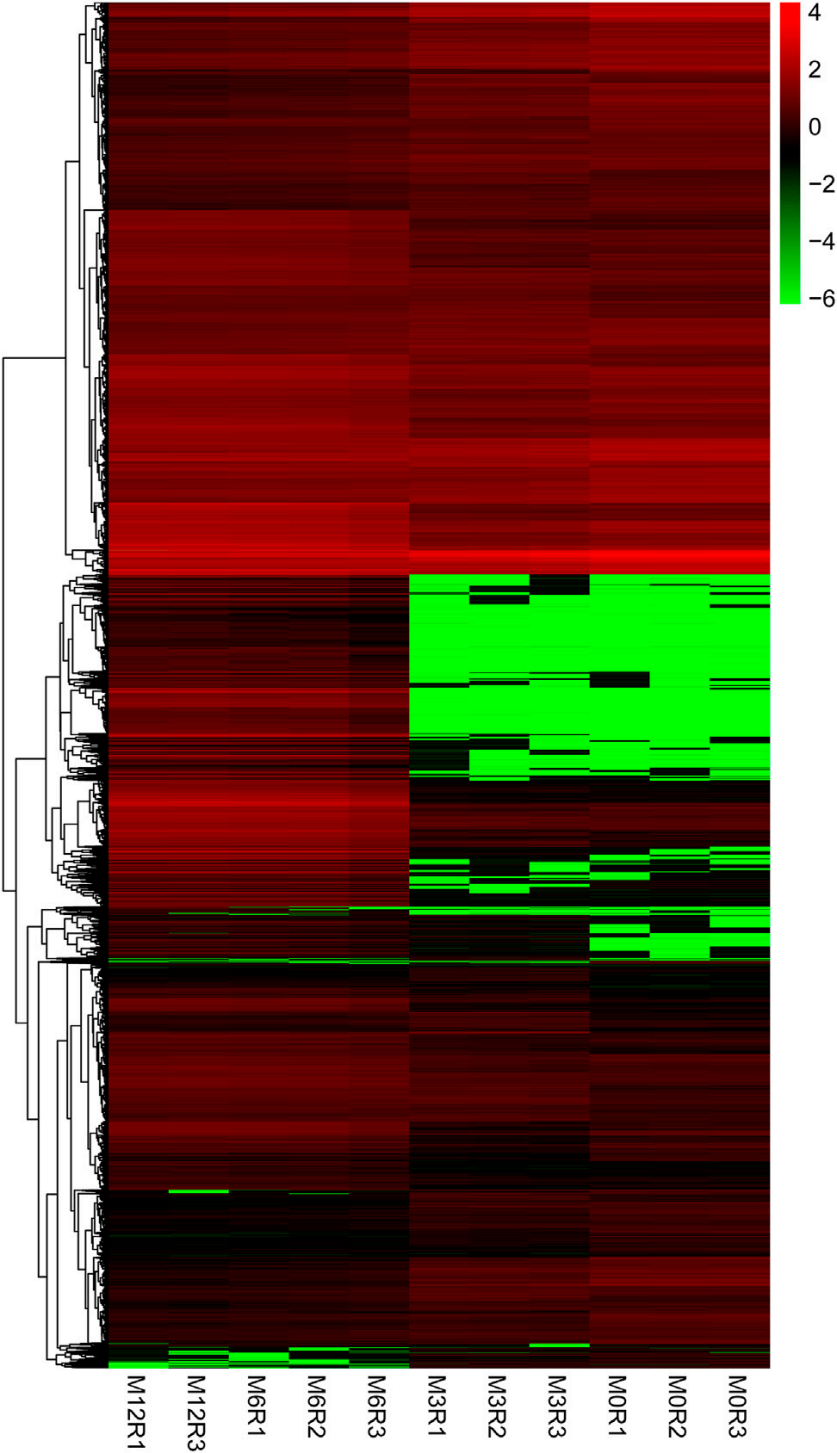
Hierarchical clustering of genes expression from M0, M3, M6, and M12 testis. Hierarchical clustering results indicated that all M0 (M0R1, M0R2, M0R3), M3 (M3R1, M3R2, M3R3), M6 (M6R1, M6R2, M6R3), M12 (M12R1, M12R3) testicular tissue samples gather together, respectively.

**FIGURE 3:**
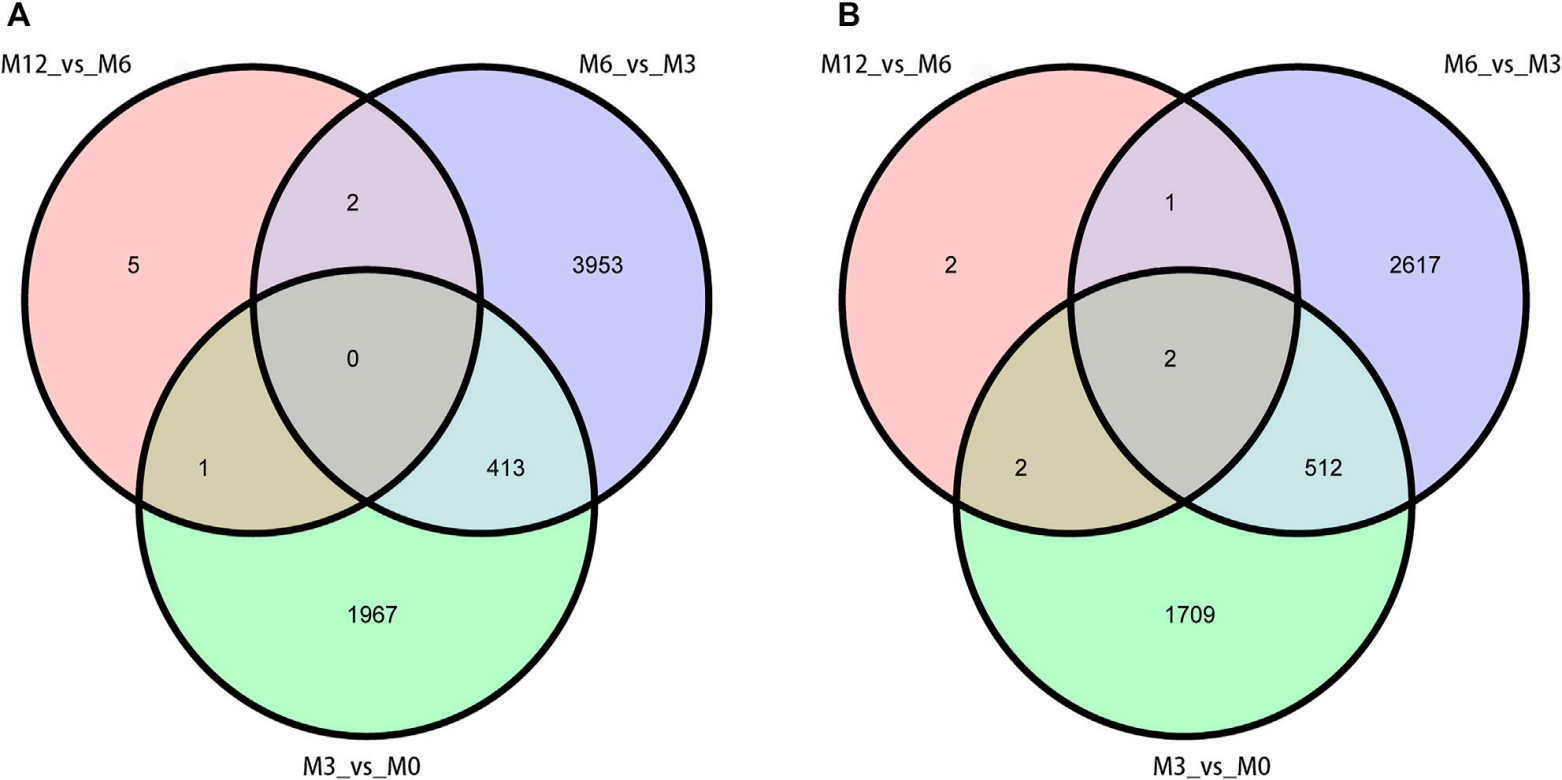
Venn diagram of DEGs obtained from three analysis groups (M3_vs_M0, M6_vs_M3, and M12_vs_M6). **(A)** up-regulation DEGs **(B)** and down-regulation DEGs.

Correlation analysis of the gene expression between paired samples showed that M12R2 correlated poorly among the M12 group. The sample named M12R2 was removed consequently to increase the reliability of the subsequent results (Supplementary Table S5 and Figure S1). Hierarchical cluster analysis of DEGs showed that the samples clustered based on condition. Only a few DEGs in M12_vs_M6 showed that there was almost no difference in gene expression between M12 and M6 (Figure 2 and Supplementary Figure S2). We identified 2,381 up- and 2,225 down-regulated, 4,368 up- and 3,132 down-regulated, and 8 up- and seven down-regulated genes in M3_vs_M0, M6_vs_M3 and M12_vs_M6, respectively (log2 FC ratio ≥1 and adjusted *p*-value <0.01). Venn maps of differentially expressed genes showed that 413 DEGs remained up-regulated and 512 DEGs remained down-regulated with aging (from M0 to M6), suggesting that these genes may be involved in testicular development (Figure 3). The number of DEGs identified indicated that testicular development spanned infancy, puberty, and sexual maturation and entered quiescence after sexual maturation.

**FIGURE 4:**
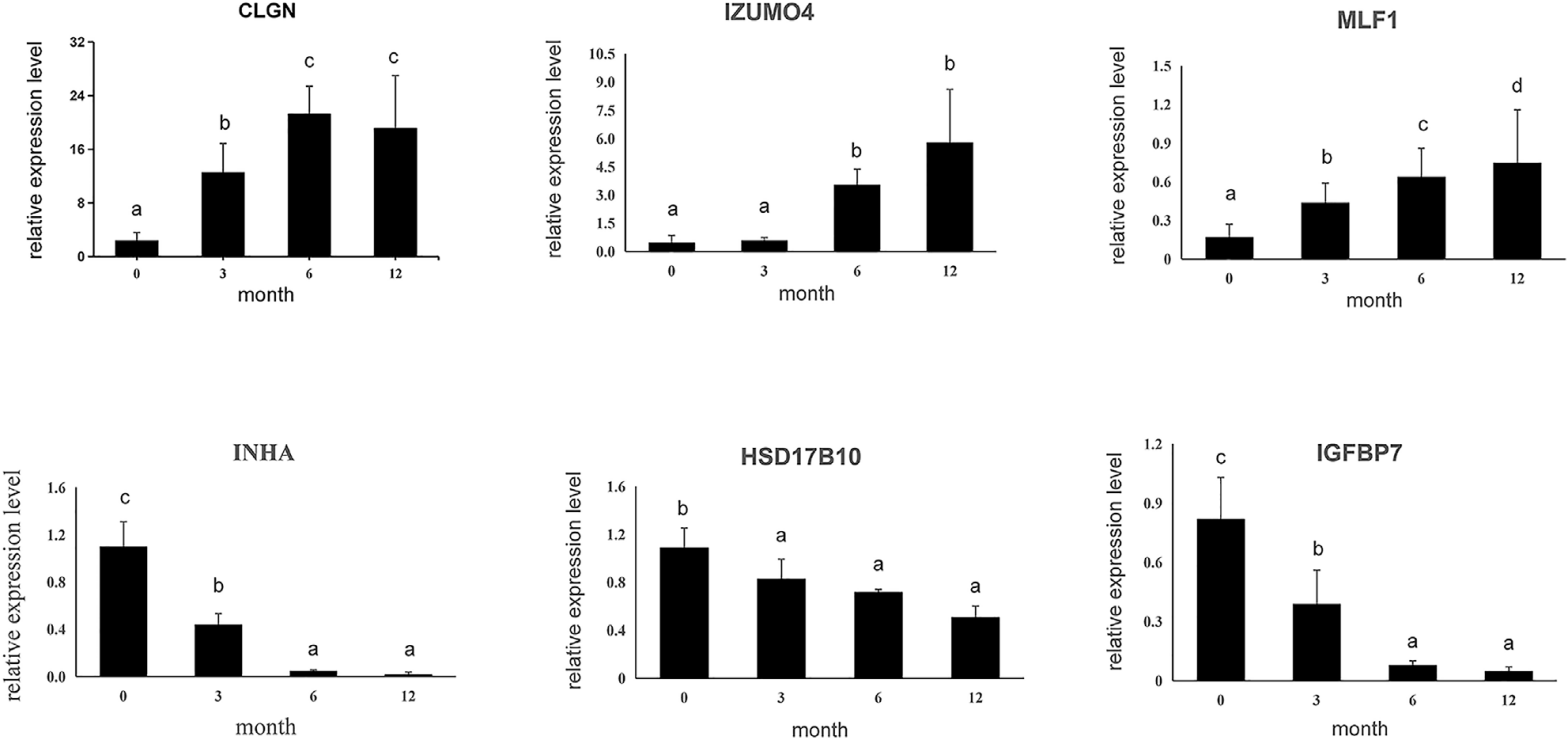
Six differential expressed genes were validated using qPCR. Data shown are the mean ± SEM. Same letter indicates that the difference is not significant; different letters represent differences. Three biological replicates were used.

Subsequently, six DEGs identified by RNA sequencing were selected to confirm expression patterns using qPCR, including three up-regulated genes (*CLGN*, *LZUMO4*, and *MLF1*) and three down-regulated genes (*HSD17B10*, *INHA,* and *IGFBP7*) in M0, M3, M6, and M12. The results showed that expression patterns of the six genes were in agreement with those of the RNA sequencing results (Figure 4), indicating that our RNA-Seq results and analyses were highly reliable.

### GO and KEGG Pathway Analysis of DEGs

**FIGURE 5:**
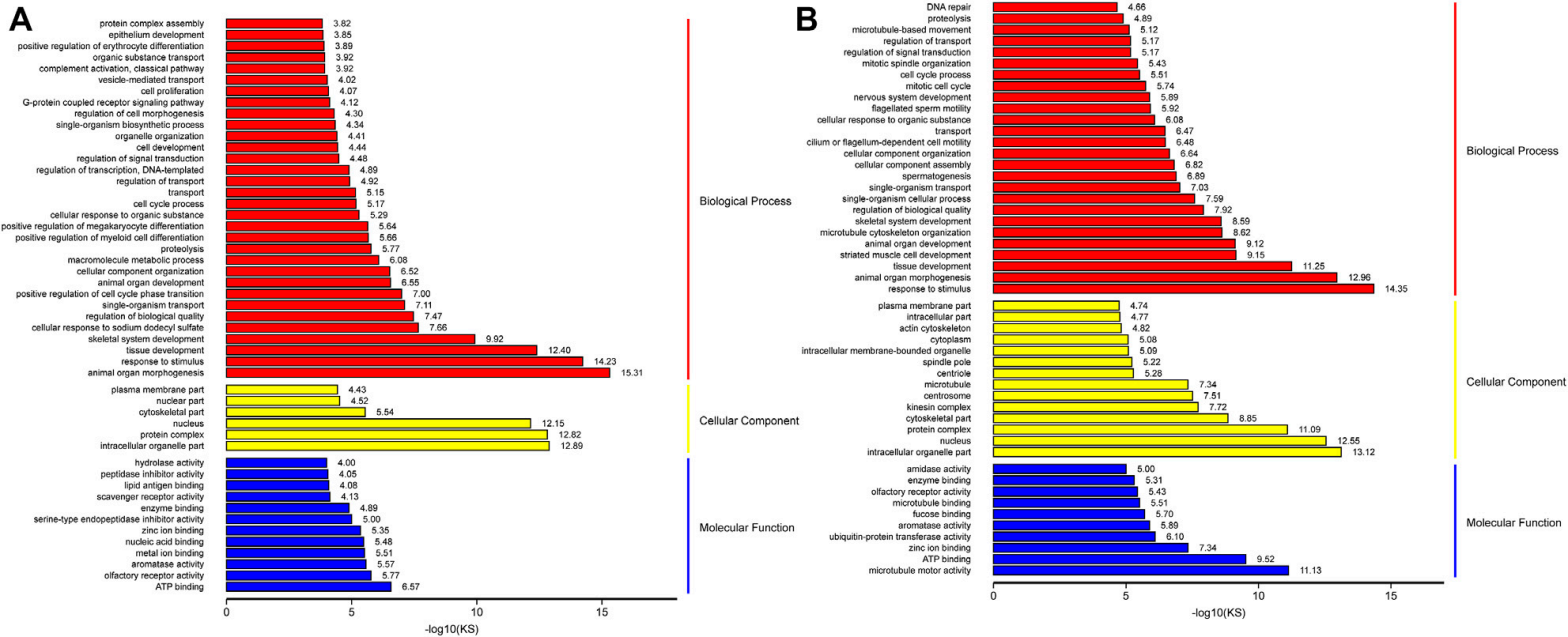
GO analysis of up-regulation genes in M3_vs_M0 analysis group **(A)** and M6_vs_M3 analysis group **(B)**.

To define the biological processes involved in M3_vsM0, we carried out a Gene Ontology analysis of the DEGs. We found 1951 out of 2,381 up-regulated genes were assigned to 6450 GO terms, namely, 4,802 biological process (BP) terms, 622 cell component (CC) terms, and 1,026 molecular function (MF) terms. We identified the 26 most significant BP terms (*p* < 9.5E-5); these terms included organ morphogenesis, response to stimulus, tissue development, skeletal system development, regulation of biological quality, single-organism transport, cell cycle control, cell development, cell proliferation and its control, metabolic processes, Leydig cell differentiation, hormone synthesis, embryo development, gland development, gonad development, androgen metabolism, cell differentiation, mitotic cell cycle, mitotic cell cycle arrest, negative regulation of mitotic anaphase, regulation of mitotic metaphase/anaphase, male sex differentiation, reproduction, stem cell differentiation, fertilization, and single fertilization. We identified the six most significant CC terms (*p* < 3.7E-5); these terms included intracellular organelle, protein complex, nucleus, cytoskeleton, and plasma membrane. Lastly, we identified the 11 most significant MF terms (*p* < 9.4E-5); these terms included ATP binding, olfactory receptor activity, aromatase activity, metal ion binding, nucleic acid binding, zinc ion binding, serine-type endopeptidase inhibitor activity, enzyme binding, scavenger receptor activity, peptidase inhibitor activity, and hydrolase activity (Figure 5A and Supplementary Table S6).

Similarly, we carried out gene ontology analysis of DEGs in M6_vs_M3 to define the key biological processes occurring in sexually mature testes. A total of 3,550 up-regulated genes were assigned to 7772 GO terms, namely, 5748 BP terms, 773 CC terms, and 1251 MF terms. We identified the 36 most significant BP terms (*p* < 8.1E-5); these terms included response to stimulus, cellular component morphogenesis, organ morphogenesis, tissue development, striated muscle cell development, microtubule cytoskeleton organization, organ development, skeletal system development, spermatogenesis, germ cell development, sperm chromatin condensation, spermatid development, male mating behavior, spermatid differentiation, penetration of zona pellucida, regulation of biological quality, single-organism cellular process, cilium or flagellum-dependent cell motility, epithelial cilium movement, cilium-dependent cell motility, cell cycle control, sperm motility, cilium assembly, single fertilization, meiosis I, epithelial cell cilial-mediated movement involved in differentiation, positive regulation of meiosis I, male meiosis, meiotic nuclear division, acrosome assembly, male meiosis I, synaptonemal complex organization, meiotic chromosome segregation, male sex differentiation, fusion of sperm to egg plasma membrane, binding of sperm to zona pellucida, sperm-egg recognition, and fertilization. We identified the 18 most significant CC terms (*p* < 7.3E-5); these terms included nucleus, intracellular organelle, protein complex, cytoskeleton, microtubule, spindle pole, microtubule cytoskeleton, cilium, synaptonemal complex, motile cilium, zona pellucida receptor complex, acrosomal vesicle, mitochondrial membrane, primary cilium, and spermatoproteasome complex. Lastly, we identified the 13 most significant MF terms (*p* < 9.5E-5); these terms included microtubule motor activity, ATP binding, zinc ion binding, microtubule-binding, aromatase activity, fucose binding, and enzyme binding (Figure 5B and Supplementary Table S6).

The number of genes within each of the aforementioned GO categories were higher for M3_vs_M6 than for M0_vs_M3, indicating that the majority of spermatogenesis-associated genes were up-regulated in sexually mature animals compared to pubertal animals. The results showed that M3 was enriched in basic metabolic processes, Leydig cell differentiation, cell cycle control, cell proliferation, cell differentiation, mitotic-related processes, androgen metabolism, and cell proliferation. M6 was enriched in spermatogenesis, germ cell development, sperm chromatin condensation, spermatid development, male mating behavior, spermatid differentiation, penetration of zona pellucida, regulation of biological quality, single-organism cellular process, cilial or flagella-dependent cell motility, epithelial cilium movement, cilium-dependent cell motility, cell cycle process, sperm motility, cilium assembly, single fertilization, and meiosis I.

KEGG analysis showed that several pathways were significantly enriched. After comparing M3 with M0, 283 pathways were enriched. We identified the nine most significantly enriched pathways (*p* < 0.05); and these pathways included focal adhesion (ko04510), HTLV-1 infection (ko05166), ECM-receptor interaction (ko04512), progesterone-mediated oocyte mature (ko04914), cell cycle (ko04110), steroid biosynthesis (ko00100), Rap I signaling pathway (ko04015), proteoglycans in cancer (ko05205), and Hippo signaling pathway (ko04390) (Supplementary Figure S3 and Table S7). After comparing M6 with M3, 283 pathways were enriched. We identified the five most significantly enriched pathways (*p* < 0.05); these pathways included focal adhesion (ko04510), ECM-receptor interaction (ko04512), glycerophospholipid metabolism (ko00564), cell cycle control (ko04110), and choline metabolism in cancer (Supplementary Figure S3 and Table S8).

**FIGURE 6:**
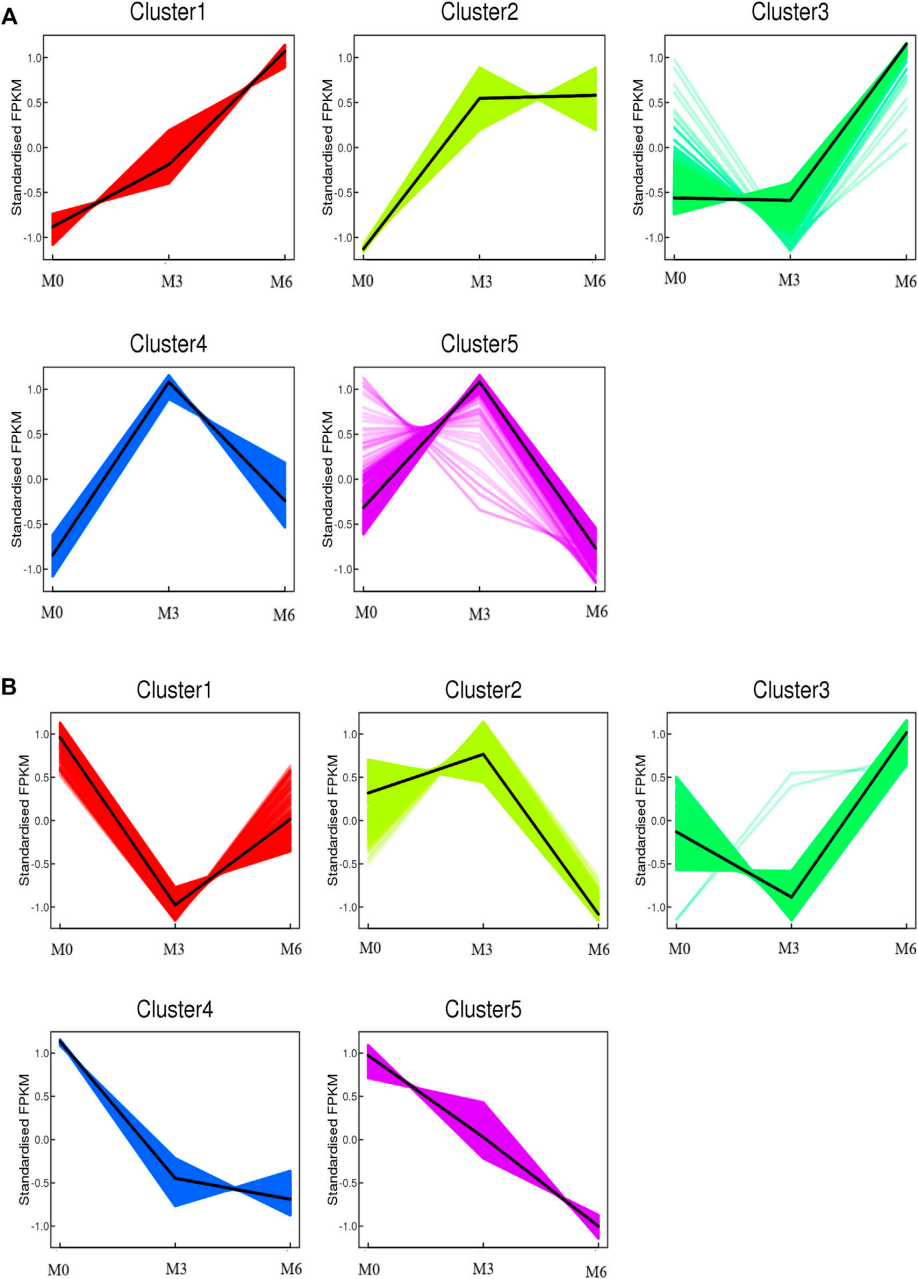
Co-expression analysis of all genes obtained from different developmental stages. **(A)** Co-expression analysis of kmean_up based on three development stages (M0, M3, M6). **(B)** Co-expression analysis of kmean_down based on three development stages (M0, M3, M6).

To further understand testis function, each gene expression model was classified on the basis of a developmental time point (Figure 6). We examined the co-expression trends of genes at three development time points, namely, M0, M3, and M6. These results indicated that testis development was very complicated. We identified 413 remained up- and 512 remained down-regulated genes involved in testis development. GO analysis showed that 306 out of 413 remaining up-regulated genes were assigned to 46 GO terms (Supplementary Figure S4). There were 23 reproduction-associated DEGs, two growth-associated DEGs, and 157 developmental process-associated DEGs (Supplementary Table S9). Similarly, 469 out of 512 remaining down-regulated genes were assigned to 55 GO terms (Supplementary Figure S4). There were 20 reproduction-associated DEGs, 46 reproductive process-associated DEGs, and 12 growth-associated DEGs, and 252 developmental process-associated DEGs (Supplementary Table S10).

### Gene Co-Expression Modules Associated With Sheep Phenotypic Traits

**FIGURE 7:**
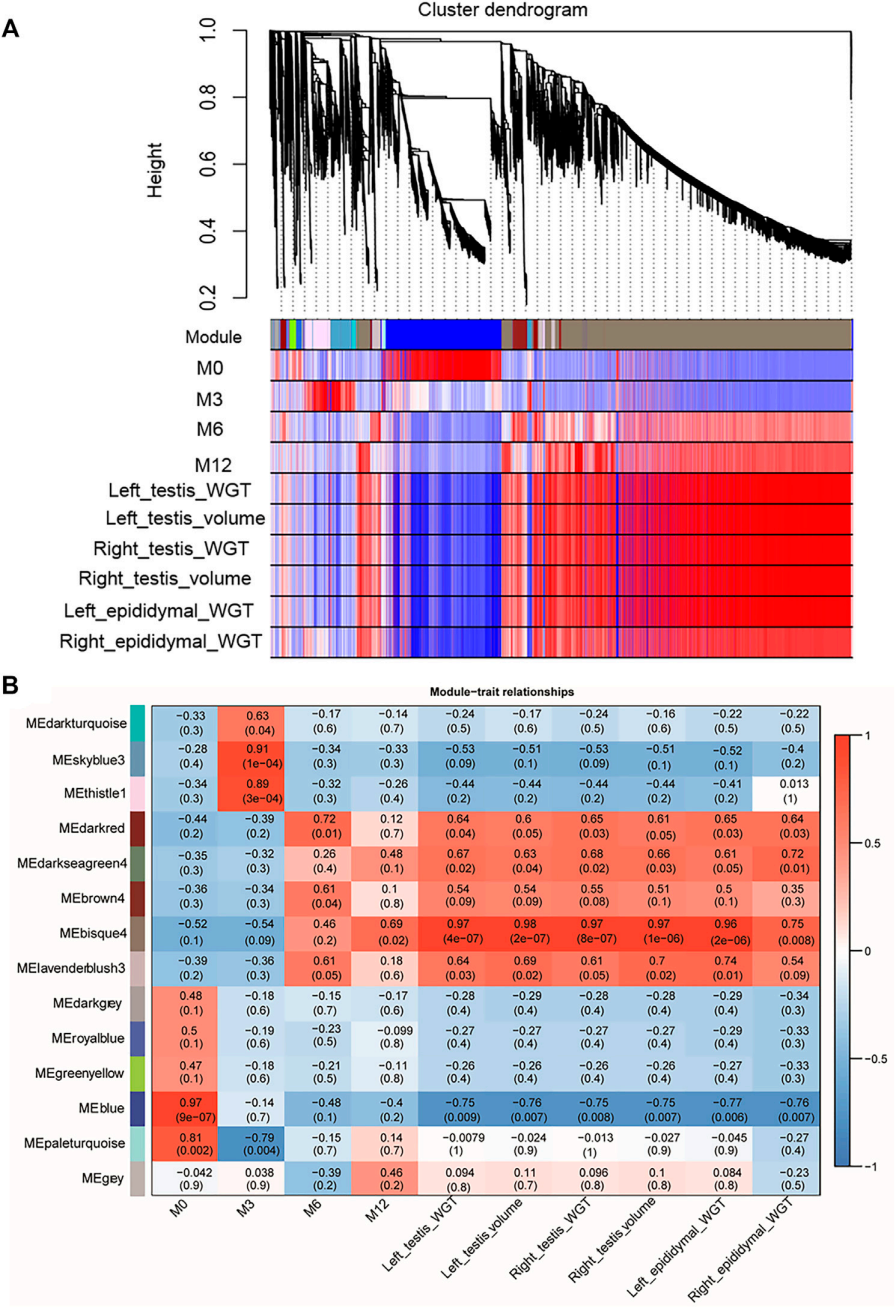
Visual representations of the gene co-expression networks and corresponding gene significant by WGCNA analysis. **(A)** The cluster dendrogram of all genes obtained from all samples. Hierarchical clustering of all genes constructed from four development phase testis of Hu sheep. The colored bars (below) directly correspond to the module (color) designation for the gene clusters. Fourteen distinct gene modules or groups of genes with high topological overlap were identified. To distinguish between modules, we designated each module with an arbitrary color; grey modules represented poorly connected genes. **(B)** The relationships between gene modules and traits (development time point and phenotypic traits) in the testis of sheep at different stages. The *y*-axis shows the 14 gene modules constructed by WGCNA. The *x*-axis shows the phenotypic traits, including Left testis weight (WGT), left testis Volume, Right testis WGT, Right testis volume, Left epididymal WGT, and Right epididymal WGT. In each cell of the table, the upper value shows the coefficient of correlation (r) between gene module and phenotypic trait, while the lower value indicates the statistical probability. Red to blue coloration of the cells indicates the transition from positive to negative correlation, as indicated by the color bar.

**FIGURE 8:**
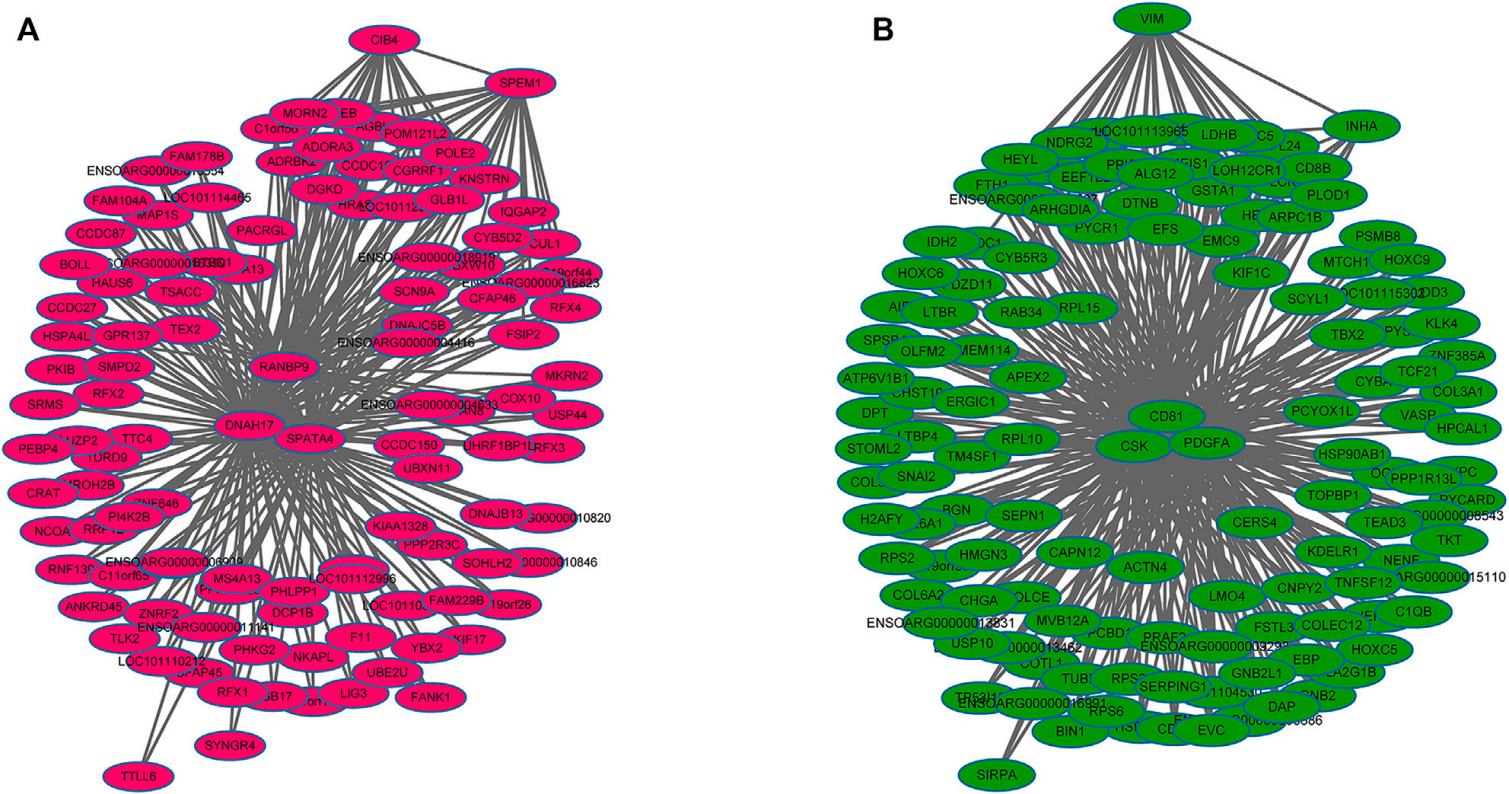
The regulation network of key genes related to testis phenotypic trait. **(A)** The regulation network of key genes that were positively in testis phenotypic traits, which indicated that some hub-genes such as *RANBP9*, *DNAH17*, *SPATA4*, *CIB4,* and *SPEM1* were identified **(B)** The regulation network of key genes that were negatively in testis phenotypic traits, which indicated that some hub-genes such as *CD81*, *CSK*, *PDGFA*, *VIM,* and *INHBA* were identified.

WGCNA analysis was used to investigate the relationship between the testis phenotypes and the gene modules at different developmental time points. We identified 14 gene modules, of which MEbisque4 and MEblue were significantly correlated with traits of our interest (correlation coefficient >± 0.75; *p* < 0.01). The MEbisque4 model, which was positively correlated with testis weight, testis volume, and epididymal weight (Figure 7), included reproductive-associated genes as follows: *DAZAP1*, *CLGN*, *PIWIL1*, *PIWIL2*, *ZPBP*, *IGF1R*, *PDGFC*, *PDGFD*, *PDGFRA*, *MYBL1*, *TSSK6*, *SPEM1*, *SPERT*, *SPACA5*, *SPAG6*, *SPATA4*, *SPATA9*, *SPATA16*, *SPATA19*, *SLC26A8*, *FSHR*, *ODF1*, *ODF2*, *ODF4*, *SPEF2*, *DDX25*, *SOX9*, *DAZL*, *LRRC6*, *IGFBPL1*, *DKKL1*, *LDHC*, *PKDREJ*, *DNAH8*, *CIB4*, *GGNBP2*, *OAZ3*, *GGN*, *ROPN1L*, *CABYR*, *SLC26A8*, and *RANBP9*. Hub genes such as *RANBP9*, *DNAH17*, *SPATA4*, *CIB4,* and *SPEM1* (Table 1) were screened among this highly related module and provided the most detailed biological information (Figure 8A). On the other hand, the MEblue model, which was negatively correlated with testis weight, testis volume, and epididymal weight (Figure 7), included reproductive-associated genes as follows: *INHBA*, *INHA*, *LOC101103417*, *OVOL2*, *LHR*, *KIT*, *PIWIL4*, *IGF1*, *PDGFA*, *PDGFB*, *PDGFRB*, *PDGFRL*, *AMH*, *INSL3*, *VEGFA*, *VEGFB*, *COL1A1*, *COL1A2*, *ODF2L*, *GATA4*, *IGFBP1*, *IGFBP3*, *IGFBP4*, *IGFBP5*, *IGFBP6*, *IGFBP7*, and *VIM*. Similarly, some hub-gene such as *CD81*, *CSK*, *PDGFA*, *VIM,* and *INHBA* (Table 1) were obtained successfully (Figure 8B).

**TABLE 1:** List of Obtained key genes from different approaches.

Screening approach	Key genes
The MEbisque4 model of WGCNA	*RANBP9*, *DNAH17*, *SPATA4*, *CIB4* and *SPEM1*
the MEblue model of WGCNA	*CD81*, *CSK*, *PDGFA*, *VIM* and *INHBA*
KEGG	*COL1A1*, *COL1A2*, *PDGFA* and *IGF1*
Continuously up-regulated key genes from 0-month-old to 6-month-old	*ODF3*, *ZPBP1*, *PKDREJ*, *MYBL1* and *PDGFA*
Continuously down-regulated key genes from 0-month-old to 6-month-old	*IGF1*, *LH*, *INSL3*, *VIM*, *AMH*, *INHBA*, *COL1A1*, *COL1A2* and *INHA*

## Discussion

Sheep are important economic livestock that can supply milk, meat, and wool, etc. The testicular size of male animals directly determines fertility and provides direct economic benefits to the sheep industry. In general, there is a complex regulatory relationship between genotype and organismal phenotypes. Transcriptomics can help to investigate the expression levels of all gene transcripts in different time stages. The genes are up-regulated or down-regulated in different levels of proteins and metabolites, which can induce phenotypic changes in the animal. Thus, understanding the regulation of genes is vitally important in the growth and development of animals, which provides insight into the biological functioning and detection of genes in some important production traits (Suravajhala et al., 2016). In this paper, we found differentially expressed genes between different growth stages by RNA-Seq in sheep testes and identified the testicular development-related genes by functional enrichment. Then, WGCNA was applied to analyze the clusters of co-regulated genes which related to testicular size to reveal genes that may play important roles in regulating testicular development. This will provide important support for breeders to select candidate genes for their future breeding programs. Here, we analyzed the genome-wide expression patterns of testes at infancy (M0), puberty (M3), sexual maturity (M6), and body maturity (M12). We identified 4,606 (2,381 up and 2,225 down), 7,500 (4,368 up and 3,132 down), and 15 (8 up and seven down) DEGs between M3 and M0, M6 and M3, M12 and M6, respectively. Because there is almost no difference between M12 and M6, the subsequent analysis in the M12_vs_M6 group was not discussed in this paper.

In the present study, we examined global gene expression in different stages of Hu sheep testis. We found that 4,606 DEGs were significantly expressed between M3 and M0. The total number of DEGs rose to 7,500 from puberty to sexual maturity and remains basically constant from sexual maturity to body maturity. According to the histological analysis of spermatogenic cell development, sperm production and testicular internal structure vary along the developmental stages. In Hu sheep, previous research has reported the structure of testicular tissue during postnatal development. According to the results of hematoxylin-eosin staining, there were only spermatogonia, Leydig cells, and Sertoli cells in 5-day-old male Hu sheep. A few primary spermatocytes appeared in the 3-month-old Hu rams and increased in the 6-month-old specimens. Spermatids and spermatozoa were found in the 9-month-old rams (Yang et al., 2018). There are three phases in the spermatogenesis of mammals including mitosis of spermatogonia, meiosis of spermatocytes, and spermiogenesis. Previous studies have shown that many changes in gene expression exist in the transition of gonocyte to spermatogonia. In yak, the DEGs were significantly expressed at this stage and gradually declined in subsequent stages (Wang et al., 2019). In mice, a similar pattern of gene expression was reported (Shima et al., 2004). Yang et al. Identified five male germ cell types including containing early primary spermatocytes, late primary spermatocytes, round spermatids, elongated spermatids, and sperm from the adult sheep testis by single-cell transcriptomic study. By knowing the changes in the transcription level of various cell types during the process of sheep spermatogenesis, Researchers found that the marker genes of early and late primary S’cytes were primarily involved in the cell cycle and related to meiosis. The other three stages were mainly related to sperm deformation such as being involved in metabolism, spermatogenesis, sperm development, and differentiation (Yang et al., 2021).

Our findings discovered that there is almost no difference between 6-month-old sheep (sexual maturity) and 12-month-old sheep (body maturity) in testicular development. Bai et al. discovered that the number of DEGs in two- vs 6-month-old testes was markedly more than the number of DEGs in six- vs-12-month-old testes in Small Tail Han Sheep. In addition, DEGs in two- vs 6-month-old testes were mainly related to sexual maturation and the pathways of multiple metabolism and biosynthesis; DEGs in six- vs-12-month-old testes were mostly related to metabolism and translation processes (Bai et al., 2017). it was believed that the sheep usually reach sexual maturity at 6 months old and the development of testes is completed before 6 months of age. Therefore, testis development-related genes have been associated with sexual maturity with decreasing expressions at later developmental stages.

From the result of GO analysis, the significantly enriched terms related to testis development and growth included spermatogenesis, sperm motility, acrosome assembly, spermatid development, synaptonemal complex, fertilization, cilium or flagellum-dependent cell motility, male meiosis I, binding of sperm to zona pellucida, and sperm-egg recognition and so on. Interestingly, in the aforementioned GO categories, some were only significantly enriched in M6_vs_M3, some genes number was higher for M6_vs_M3 than M3_vs_M0, indicating that the majority of genes involved in spermatogenesis-associated or sexual maturity-associated processes were up-regulated in sexually mature sheep. In this regard, these terms were important for sexual maturation and required further analysis.

KEGG analysis showed several shared pathways between M3/M0 and M6/M3, such as focal adhesion (ko04510), ECM-receptor interaction (ko04512), and cell cycle control (ko04110). These pathways are all associated with structural traits pertaining to spermatogenesis. The focal adhesion (ko04510) and ECM-receptor interaction (ko04512) were associated with male germ cells development, differentiation, and maturation. Focal adhesion kinase (FAK) may be involved in Sertoli–Sertoli, and Sertoli–germ cell adhesion, as well as germ cell transport during spermatogenesis because of cell junction restructuring (Siu et al., 2009; Zhang et al., 2013). The cell cycle (ko04110) was related to the male germ cells division and proliferation (Li and Qian, 2017). We also identified several reproduction-associated DEGs (*p* < 0.05), including *COL1A1*, *COL1A2*, *PDGFA,* and *IGF1* (Table 1), indicating that these pathways are crucial for testis development (Supplementary Table S7, S8). These data indicated that the majority of genes involved in testis development promote testicular development, sexual maturation, and spermatogenesis by participating in complex biological events.

Functional analysis showed persistent changes in 413 up- and 512 down-regulated genes that were involved in reproduction, reproductive processes, developmental processes, and growth and hormone secretion. Several continuously up-regulated genes, such as *ODF3*, *ZPBP1*, *PKDREJ*, *MYBL1,* and *PDGFA* (Table 1) presumed to be functionally important for sperm maturation and acrosome reaction in pubertal animals (Supplementary Table S9). In addition, several continuously down-regulated genes, such as *IGF1*, *LH*, *INSL3*, *VIM*, *AMH*, *INHBA*, *COL1A1*, *COL1A2,* and *INHA* (Table 1), indicated that the high transcriptomics activity of these genes was critical for immature testicular development at newborn-infant stages (Supplementary Table S10).

To further investigate the key genes involved in spermatogenesis and testis development, we explored the relationships among all gene models obtained from different development stages and phenotypic traits. Results from WGCNA showed that two out of 14 models were significantly associated with testis phenotypic traits. The bisque4 model, which was positively associated with testis phenotypic traits and several hub-genes (*RANBP9*, *DNAH17*, *SPATA4*, *CIB4,* and *SPEM1*) (Table 1), was found to play an important core function in testis maturation in adult sheep. As expected, the differentially expressed genes are consistent with the enlargement of testis mass. The blue model negatively associates with testis phenotypes, and five genes (*CD81*, *CSK*, *PDGFA*, *VIM,* and *INHBA*) (Table 1) have been shown to control testis size and development. More importantly, all five of these genes were differentially expressed at different phases and transcriptional activity of these genes decreased with increasing age and testis weight, suggesting that they played crucial roles in testis development.

There were six genes selected from two screening approaches and over including *IGF1*, *VIM*, *COL1A1*, *COL1A2*, *PDGFA,* and *INHBA*. We summarized these genes functions in previously published work in order to better understand the roles of key genes above. Previous data obtained from mice and humans showed that insulin-like growth factor-I (*IGF1*) and luteinizing hormone (LH receptor) were key regulators of reproductive functions. *IGF1*, which is expressed in mouse developing testes, regulates somatic cell proliferation and facilitates growth and development (Vannelli et al., 1988; Villalpando et al., 2008). Furthermore, it was speculated that *IGF1* played a key role in the regulation of Sertoli cell number, testis size, and daily sperm output (Griffeth et al., 2014). We also identified these two genes in sheep; their expression peaked in newborns (*p* < 0.05) and gradually decreased with age, suggesting that *IGF-1* and *LHR* have key roles in somatic cell proliferation, Sertoli cell differentiation, and individual growth and development. A related study also found that *VIM* (vimentin) and *AMH* (anti-Mullerian hormone) were molecular markers of testicular development. They were highly expressed in immature testicles and weakly in adult testicles (Holt et al., 2010). The literature suggested that *COL1A1* (collagen type I alpha one chain) and *COL1A2* (collagen type I alpha two chain) are associated with type A spermatogonia and played potential roles in mediating the detachment and migration of germ cells during spermatogenesis (He et al., 2005). Platelet-derived growth factor (*PDGF*), a key regulator of connective tissue cells during embryogenesis and pathogenesis, was one of the locally produced growth factors that mediate testicular cell-cell interactions (Mariani et al., 2002). It was reported that adult testicular dysgenesis was triggered in *Inhba* conditional knockout mice, which was explained by the fact that cross-talk between Leydig cells and germ cells was disrupted (Sun et al., 2010). Therefore, we speculated that *INHBA* (inhibin subunit beta A) was critical for physical interactions between germ and somatic cells from immature testicular development in the ovine newborn-infant phase.

Furthermore, we have concentrated on the expression levels of several spermatogenesis genes, such as *CLGN, SPEF2,* and *DAZL.* They all peaked at 6 months old revealing that these genes had important roles in ovine sexual maturation. For example, Male mice deficient in the calmegin (*CLGN*) gene showed impaired sperm migration into the oviduct (Yamaguchi et al., 2006). The Sperm flagella 2 (*SPEF2*) is essential for normal sperm tail development and male fertility. The loss of *SPEF2* in mice resulted in spermatogenesis defects and primary ciliary dyskinesia, which decreased the number of elongating spermatids and disrupted the formation of the sperm tail. Thus, it was one of the determinants of sperm motility in bulls, and evidence indicated that there was a positive correlation between the level of *SPEF2* expression and male fertility (Fischer et al., 2015). The Deleted in AZoospermia Like (*DAZL*), a novel germ cell marker, was found to exist in SNP and to associate with semen quality in human and pig. The loss of *the DAZL* gene led to the decrease of germ cell numbers before birth, and the loss of *DAZL* protein led to azoospermia or severe oligospermia (Ma et al., 2013; Zhu et al., 2014).

We have already identified many DEGs associated with testis development in testis size and spermatogenesis, and we validated the expression pattern of some DEGs by qPCR. Further studies and experiments are warranted to explain the regulatory mechanism of these genes. To investigate the interaction between RNA and proteins and the regulatory mechanism leads to revealing the critical factor of functional alterations. This would provide valuable insights into animal breeding.

In conclusion, this study described a genome-wide comparison among ovine transcriptome derived from the M0, M3, M6, and M12 sheep testes by RNA-Sequencing. A number of key genes associated with reproduction were identified, which could be defined as candidate genes controlling testis development and spermatogenesis. The current study outlined the detailed transcriptome changes in ovine testes at different developmental stages and provided new insights on the differently expressed genes that associate with spermatogenesis.

## Data Availability

All 11 high-throughput RNA sequencing data analyzed in this study are deposited in Sequence Read Archive (SRA) database in NCBI under accession number PRJNA763055 (https://www.ncbi.nlm.nih.gov/sra/PRJNA763055).
