# Supplementary material for: Identification of Key Genes Related to Postnatal Testicular Development Based on Transcriptomic Data of Testis in Hu Sheep
Source: Front Genet. 2022 Jan 25;12:773695. doi: 10.3389/fgene.2021.773695 (PMC8822165; doi:10.3389/fgene.2021.773695)
Supplement: Supplementary file 4 [file DataSheet2.docx]

**Supplementary information for:**

**Identification of key genes related to testicular development based on transcriptomic data of testis in Hu sheep**

**Supplementary Figure S1**.Cluster analysis of gene expression of samples.


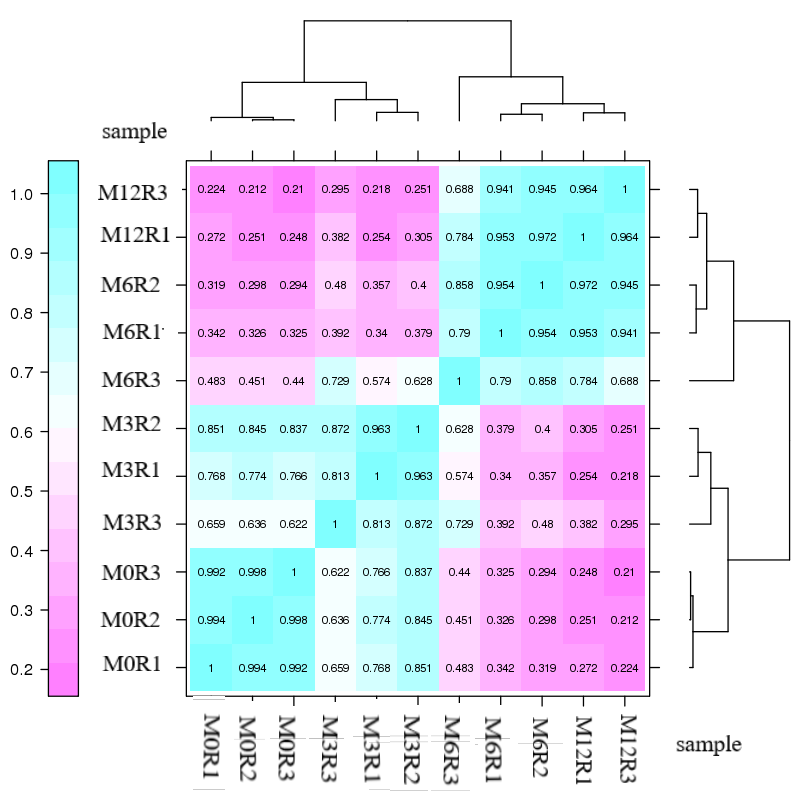


M0, M3, M6 and M12 testicular tissues gather toghter, respectively. Cluster analysis results of gene expression of samples indicated that the correlation coefficient between different stages was relatively low.

**Supplementary Figure S2**.Cluster analysis of gene expression of samples.


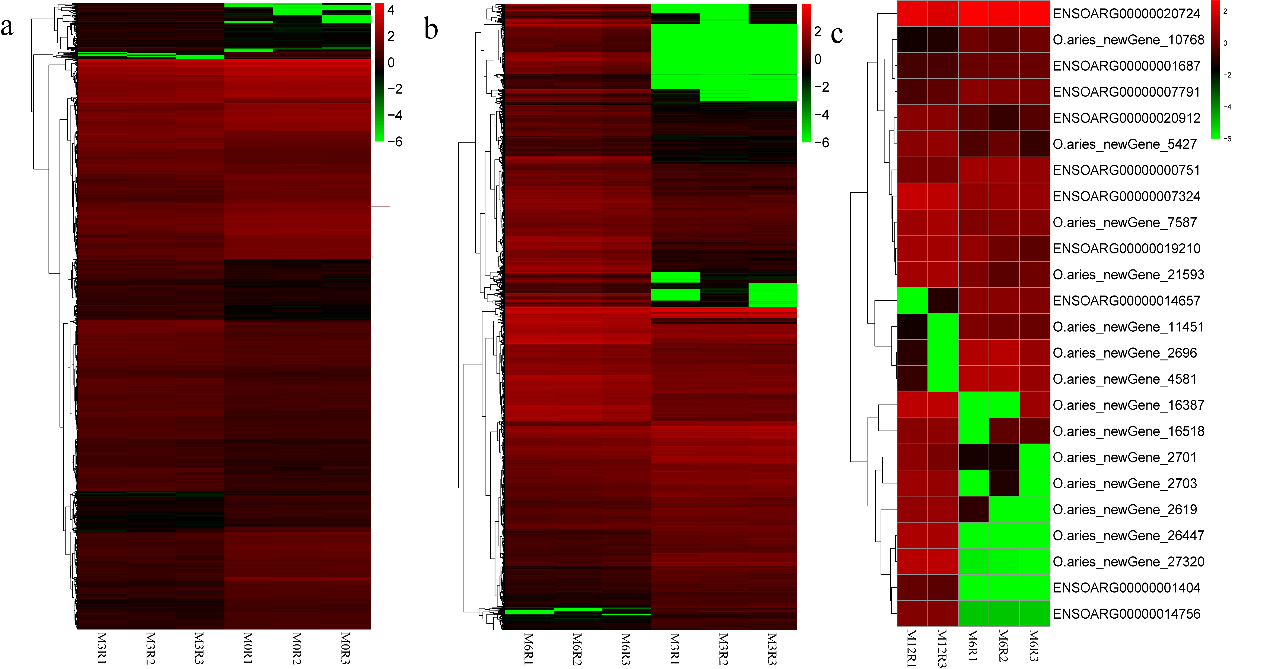


(a) Cluster analysis of gene expression between M3 and M0 samples. (b) Cluster analysis of gene expression between M6 and M3 samples. (c) Cluster analysis of gene expression between M12 and M6 samples. Cluster analysis results of gene expression indicated that all samples were gather corresponding to month ages, respectively.

**Supplementary Figure S3**. KEGG classification of DEGs in different developmental stages


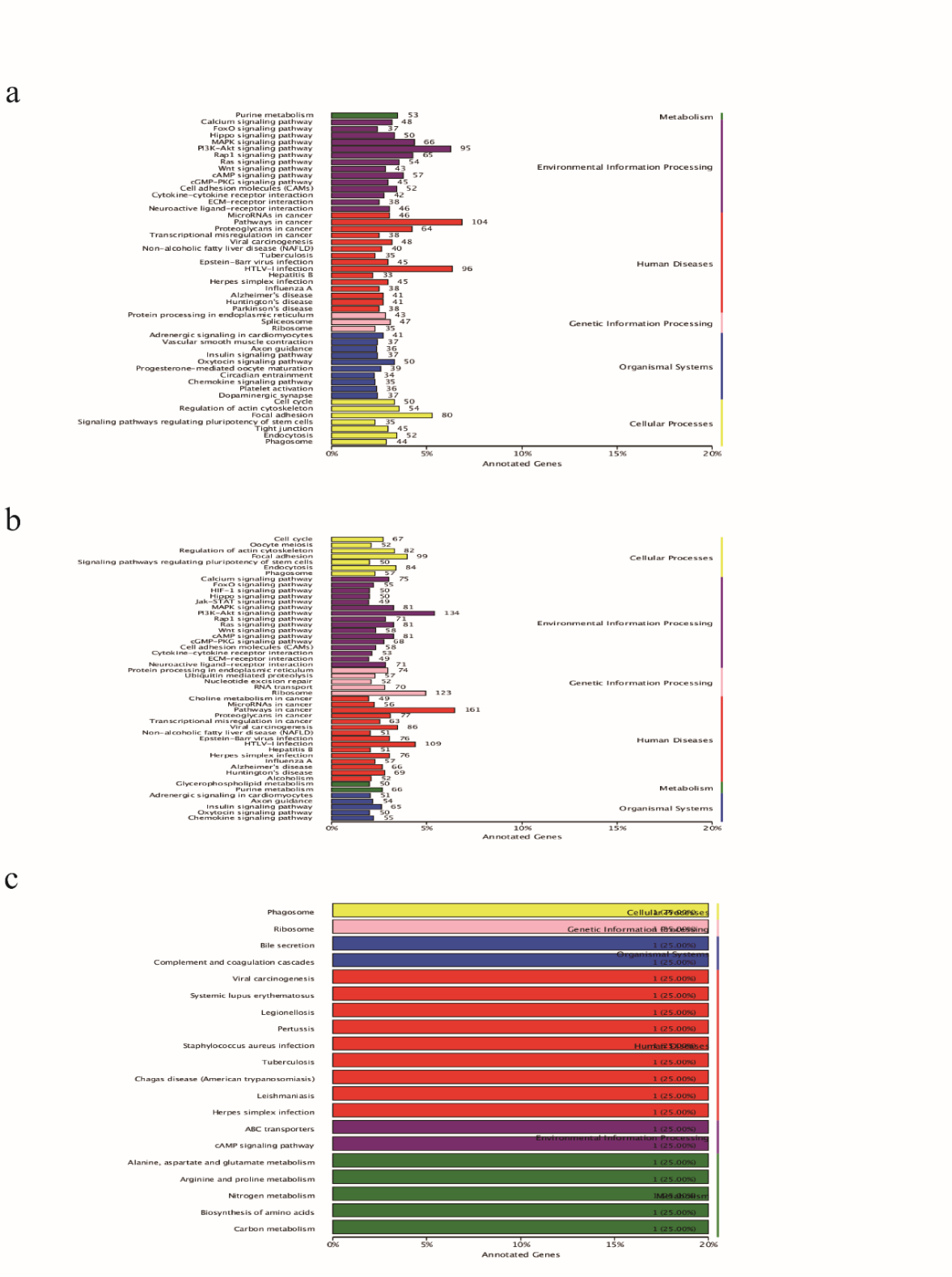


The KEGG enrichment analysis results of DEG in M3 versus M0 (a) and M6 versus M3 (b) in combined expression analysis

**Supplementary Figure S4**. 413 up- and 512 down- regulation genes GO analysis


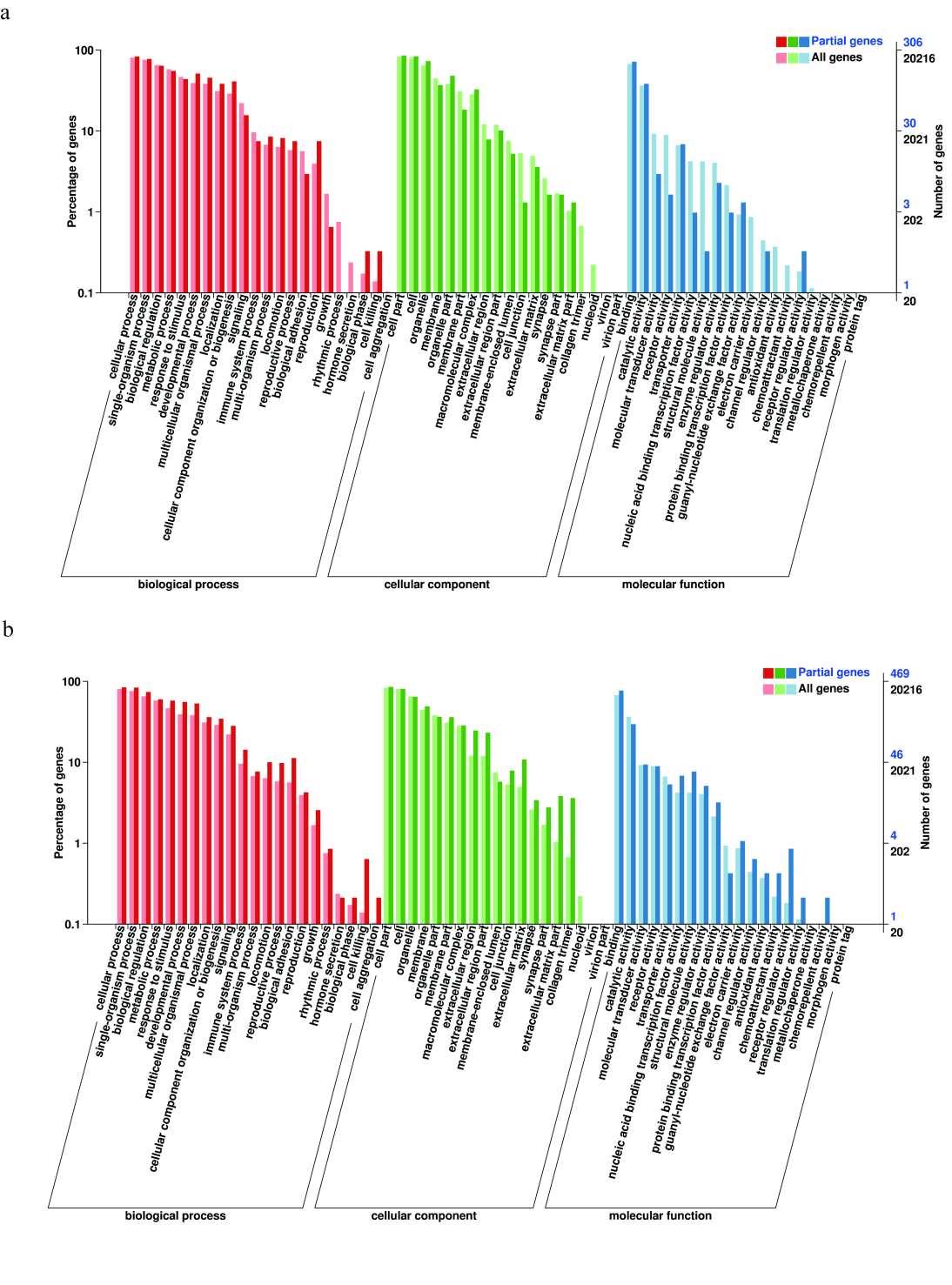


GO classification of 413 up-regulation genes (a) and 512 down- regulation genes(b)

**Supplementary** **Table S1** Primers for real-time quantitative PCR of differential gene

| Gene name | Accesstion numbers | Primer sequence(5’→3’) | | Product Size (bp) | Annealing  Temperature (°C) |
| --- | --- | --- | --- | --- | --- |
| *HSD17B10* | XM_004022064 | Forward | GTGTGGATGTGGCAGTCAAC | 127 | 60 |
|  |  | Reverse | AAGGTGCCCATGAGATTCAC |  |  |
| *IZUMO4* | XM_015095664 | Forward | CCGTGTACCAGAGGATGGAT | 116 | 60 |
|  |  | Reverse | GATGGCATTCTGGATGAGGT |  |  |
| *CLGN* | XM_015101510 | Forward | CGCCGATGATCGATAACCCT | 126 | 60 |
|  |  | Reverse | CAGCGGTCTGCTACTTCCTT |  |  |
| *PGAM*2 | XM_004018189 | Forward | TGTGGTTGGTTCGATGCAGA | 264 | 60 |
|  |  | Reverse | CTCCTCCCCGTGTTTAGCAG |  |  |
| *MLF1* | XM_012100603 | Forward | TGGCAGTTTTGGTGGTATGC | 239 | 60 |
|  |  | Reverse | CCTTTATTCCTCCTGGAGCCC |  |  |
| *IGFBP*7 | NM_001145181 | Forward | GAGCAAGGTCCTTCCATCGT | 246 | 60 |
|  |  | Reverse | AAGAGGAGATACCAGCACCCA |  |  |
| *IGFBP*6 | EU862545 | Forward | GGTGTCGGCGGGTTGTC | 355 | 60 |
|  |  | Reverse | TTGTACGCCCCCAGAATTGG |  |  |
| *SETD*9 | XM_004016970 | Forward | ATTTGATGTGCCTGCAGCTT | 121 | 60 |
|  |  | Reverse | TTCAATGTCCCTGAGTGCAA |  |  |
| *VIM* | XM_004014247 | Forward | GGGACAAGATAGCTACCCGC | 260 | 60 |
|  |  | Reverse | GTCGAAGGCCCGTGTAAAGA |  |  |
| *MEX3C* | XM_015103552 | Forward | TGCTCCGGAGAAAGAAGGTT | 310 | 60 |
|  |  | Reverse | GTCTGACCCGGCAGATTAGG |  |  |
| *INHA* | NM_001308579 | Forward | AGAGCCGCCCTCAATATCTC | 283 | 60 |
|  |  | Reverse | GGTTGGGCACCATCTCATACT |  |  |
| *COL1A2* | XM_004007726 | Forward | TGGACCAATGGGGTTGATGG | 272 | 60 |
|  |  | Reverse | CCCTAATGCCCTTGAAGCCA |  |  |
| *ACTB* | NM_001009784 | Forward | CCTGCGGCATTCACGAA | 134 | 60 |
|  |  | Reverse | GCGGATGTCGACGTCACA |  |  |

**Supplementary** **Table S2.** Obtain the high-quality clean reads

| Sample | Raw Reads | Clean Reads |
| --- | --- | --- |
| M0R1 | 22603572 | 22592276 |
| M0R2 | 22259392 | 22248268 |
| M0R3 | 23846558 | 23836640 |
| M3R1 | 22745201 | 22730836 |
| M3R2 | 22073870 | 22062839 |
| M3R3 | 21978949 | 21967965 |
| M6R1 | 21648924 | 21638105 |
| M6R2 | 21719049 | 21708195 |
| M6R3 | 28164621 | 28024500 |
| M12R1 | 45608852 | 45437671 |
| M12R2 | 24655381 | 24643069 |
| M12R3 | 26396881 | 26305361 |

**Supplementary** **Table S3.** Q30 and GC content of RNA-seq

| Sample | ReadSum | BaseSum | GC(%) | N(%) | Q30(%) |
| --- | --- | --- | --- | --- | --- |
| M0R1 | 22592276 | 6.62E+09 | 55.34 | 0.01 | 89.10 |
| M0R2 | 22248268 | 6.52E+09 | 56.84 | 0.01 | 88.83 |
| M0R3 | 23836640 | 7.01E+09 | 57.15 | 0.01 | 86.88 |
| M3R1 | 22730836 | 6.61E+09 | 55.05 | 0.01 | 89.82 |
| M3R2 | 22062839 | 6.32E+09 | 53.44 | 0.01 | 89.87 |
| M3R3 | 21967965 | 6.34E+09 | 53.30 | 0.01 | 88.66 |
| M6R1 | 21638105 | 6.42E+09 | 52.15 | 0 | 89.24 |
| M6R2 | 21708195 | 6.44E+09 | 53.00 | 0 | 89.36 |
| M6R3 | 28024500 | 8.30E+09 | 52.75 | 0 | 88.49 |
| M12R1 | 45437671 | 1.35E+10 | 52.37 | 0 | 89.15 |
| M12R2 | 24643069 | 7.22E+09 | 52.96 | 0 | 87.15 |
| M12R3 | 26305361 | 7.81E+09 | 53.23 | 0 | 88.82 |

**Supplementary** **Table S4.** Mapping of clean reads on the sheep genome

| Sample | Total Reads | Mapped Reads | Uniq Mapped Reads | Multiple Map Reads | Reads Map to '+' | Reads Map to '-' |
| --- | --- | --- | --- | --- | --- | --- |
| M0R1 | 45184552 | 29,784,553 (65.92%) | 28,410,016 (62.88%) | 1,374,537 (3.04%) | 14,504,455 (32.10%) | 14,543,036 (32.19%) |
| M0R2 | 44496536 | 27,519,561 (61.85%) | 26,132,310 (58.73%) | 1,387,251 (3.12%) | 13,360,725 (30.03%) | 13,381,508 (30.07%) |
| M0R3 | 47673280 | 27,996,071 (58.72%) | 26,538,557 (55.67%) | 1,457,514 (3.06%) | 13,576,315 (28.48%) | 13,595,579 (28.52%) |
| M3R1 | 45461672 | 30,328,467 (66.71%) | 29,132,176 (64.08%) | 1,196,291 (2.63%) | 14,798,644 (32.55%) | 14,801,146 (32.56%) |
| M3R2 | 44125678 | 29,510,722 (66.88%) | 28,262,254 (64.05%) | 1,248,468 (2.83%) | 14,360,240 (32.54%) | 14,385,188 (32.60%) |
| M3R3 | 43935930 | 28,556,405 (65.00%) | 27,220,087 (61.95%) | 1,336,318 (3.04%) | 13,854,290 (31.53%) | 13,859,095 (31.54%) |
| M6R1 | 43276210 | 32,513,302 (75.13%) | 31,764,931 (73.40%) | 748,371 (1.73%) | 16,123,649 (37.26%) | 16,093,216 (37.19%) |
| M6R2 | 43416390 | 32,261,454 (74.31%) | 31,549,418 (72.67%) | 712,036 (1.64%) | 16,011,892 (36.88%) | 15,979,783 (36.81%) |
| M6R3 | 56049000 | 41,110,731 (73.35%) | 40,123,163 (71.59%) | 987,568 (1.76%) | 20,356,053 (36.32%) | 20,336,161 (36.28%) |
| M12R1 | 90875342 | 68,004,868 (74.83%) | 66,429,323 (73.10%) | 1,575,545 (1.73%) | 33,767,171 (37.16%) | 33,685,339 (37.07%) |
| M12R2 | 49286138 | 32,818,920 (66.59%) | 31,547,253 (64.01%) | 1,271,667 (2.58%) | 16,039,440 (32.54%) | 16,072,425 (32.61%) |
| M12R3 | 52610722 | 38,434,182 (73.05%) | 37,581,296 (71.43%) | 852,886 (1.62%) | 19,085,472 (36.28%) | 19,042,793 (36.20%) |

Total mapped%= total mapped/ clean reads; total mapped: clean reads mapped on the sheep genome;

Unique mapped%=unique mapped/ total mapped; unique mapped: reads mapped only one region to the reference genome;

Multiple mapped%= multiple mapped/ total mapped; multiple mapped: reads mapped more than one region to the reference genome.

**Supplementary** **Table S5.** Correlation analysis of the gene expression between paired samples.

|  | M12R1 | M12R2 | M12R3 | M6R1 | M6R2 | M6R3 | M3R1 | M3R2 | M3R3 | M0R1 | M0R2 | M0R3 |
| --- | --- | --- | --- | --- | --- | --- | --- | --- | --- | --- | --- | --- |
| M12R1 | 1 | 0.2064 | 0.9638 | 0.9534 | 0.9724 | 0.7841 | 0.2536 | 0.3049 | 0.3816 | 0.2723 | 0.2509 | 0.2483 |
| M12R2 | 0.2064 | 1 | 0.165 | 0.1789 | 0.2303 | 0.3262 | 0.3003 | 0.342 | 0.427 | 0.2805 | 0.2721 | 0.2682 |
| M12R3 | 0.9638 | 0.165 | 1 | 0.9409 | 0.945 | 0.6878 | 0.2183 | 0.2513 | 0.2948 | 0.2244 | 0.2124 | 0.2103 |
| M6R1 | 0.9534 | 0.1789 | 0.9409 | 1 | 0.9541 | 0.7904 | 0.3404 | 0.3786 | 0.3921 | 0.3425 | 0.3255 | 0.3253 |
| M6R2 | 0.9724 | 0.2303 | 0.945 | 0.9541 | 1 | 0.8578 | 0.3574 | 0.4001 | 0.4797 | 0.319 | 0.2985 | 0.2935 |
| M6R3 | 0.7841 | 0.3262 | 0.6878 | 0.7904 | 0.8578 | 1 | 0.5739 | 0.628 | 0.729 | 0.483 | 0.4508 | 0.4403 |
| M3R1 | 0.2536 | 0.3003 | 0.2183 | 0.3404 | 0.3574 | 0.5739 | 1 | 0.9626 | 0.8128 | 0.7683 | 0.7744 | 0.766 |
| M3R2 | 0.3049 | 0.342 | 0.2513 | 0.3786 | 0.4001 | 0.628 | 0.9626 | 1 | 0.8715 | 0.8509 | 0.8447 | 0.8368 |
| M3R3 | 0.3816 | 0.427 | 0.2948 | 0.3921 | 0.4797 | 0.729 | 0.8128 | 0.8715 | 1 | 0.6587 | 0.6356 | 0.6216 |
| M0R1 | 0.2723 | 0.2805 | 0.2244 | 0.3425 | 0.319 | 0.483 | 0.7683 | 0.8509 | 0.6587 | 1 | 0.9944 | 0.9916 |
| M0R2 | 0.2509 | 0.2721 | 0.2124 | 0.3255 | 0.2985 | 0.4508 | 0.7744 | 0.8447 | 0.6356 | 0.9944 | 1 | 0.9983 |
| M0R3 | 0.2483 | 0.2682 | 0.2103 | 0.3253 | 0.2935 | 0.4403 | 0.766 | 0.8368 | 0.6216 | 0.9916 | 0.9983 | 1 |

**Supplementary Table S7** DEGs involed in significantly pathway in M3 vs M0 analysis group

| Pathway | ko_id | Number of gene | *P*-value | Gene name |
| --- | --- | --- | --- | --- |
| Focal adhesion | ko04510 | 80 | 4.2E-07 | RELN；KDR；TNXB；CAV2；MAPK8；COL1A2；PGF；ENSOARG00000000300；MAPK9；COL5A1；CCND3；ACTB；FLNC；ACTN2；CHAD；COL1A1；ENSOARG00000005037；FLNA；VEGFB；ITGA3；ACTN4；TNC；COL27A1；COL4A1；PDGFA；ARHGAP5；ITGB6；PIK3CB；HRAS；LAMA3；ENSOARG00000007807；PARVA；VEGFA；ITGB4；C-JUN；VASP；CCND2；MRCL3；COMP；ITGA7；ERBB2；FLT1；CRK；COL6A1；COL6A2；ENSOARG00000013462；LAMA5；BRAF；EGFR；RHOA；COL5A3；COL6A6；COL6A5；MYL9；IGF1；ITGA5；COL5A2；LOC101106246；COL3A1；ILK；CCND1；THBS4；PAK3；ACTB；COL11A1；COL6A3；PARVG；FN1；COL2A1；THBS1；COL4A3；ITGA10；SHC4；ACTN1 |
| HTLV-I infection | ko05166 | 96 | 8.8E-05 | ATF2；TRRAP；ENSOARG00000000431；ENSOARG00000000058；ENSOARG00000001165；BCL2L1；C-FOS；TGFB3；ANAPC2；CCND3；ADCY9；ENSOARG00000002962；DQA；VDAC3；CDC26；LOC101120622；CDKN2C；CDK4；ZFP36；PDGFA；ATR；CDC26；ANAPC4；PIK3CBE2F2；HRAS；MAP3K1；ADCY8；TGFB1；ENSOARG00000007807；MYC；WNT4；RB1；LTBR；FZD2；PRKX；CD3E；E2F3；ENSOARG00000009727；CALR；C-JUN；CCND2；RAN；WNT9B；TGFB2；CDC27；CHEK1；APC2；ATM；WNT5B；IL1R1；RRAS；ENSOARG00000013462；PTTG1；CDKN1A；CHUK；ADCY2；ENSOARG00000015002；LOC101118476；WNT5A；ENSOARG00000015485；MAD2L1；DQA1；KAT2B；LOC101120118；CDC23；LOC101105860；IL2RG；POLD2；APC；OVAR-DQA2；EGR1；HLA-DRA；FZD1；RELA；NRP1；CCND1；MYBL1；ANAPC11；EP300；LOC101114226；RANBP1；KAT2A；TNFRSF13C；ENSOARG00000018802；ENSOARG00000019064；NFATC4；WNT2B；WNT6；XPO1；ENSOARG00000020186；CDC20；TNFRSF1A； |
| ECM-receptor interaction | ko04512 | 38 | 0.0003 | RELN；TNXB；COL1A2；ENSOARG00000002032；ENSOARG00000000300；COL5A1；CHAD；COL1A1；ENSOARG00000005037；ITGA3；TNC；COL27A1；COL4A1；ITGB6；LAMA3；HSPG2；ITGB4；COMP；ITGA7；COL6A1；COL6A2；LAMA5；COL5A3；COL6A6；COL6A5；ITGA5；COL5A2；COL3A1；SDC1；THBS4；COL11A1；CD44；COL6A3；FN1；COL2A1；THBS1；COL4A3；ITGA10 |
| Progesterone-mediated oocyte maturation | ko04914 | 39 | 0.0012 | PKMYT1；CDC25B；MAPK8；MAPK9；RPS6KA6；ANAPC2；ADCY9；CDC26；HSPCA；CPEB4；CDC2；LOC101113385；CCNB1；CDC26；PDE3B；ANAPC4；PIK3CB；ADCY8；ENSOARG00000007807；CPEB2；PRKX；HSP90AB1；CCNB3；GNAI2；CDC27；ENSOARG00000013462；BRAF；CCNA2；BUB1；ADCY2；RPS6KA3；MAD2L1；IGF1；CDC23；CPEB3；ANAPC11；ENSOARG00000020186；CCNB2；ENSOARG00000000431 |
| Cell cycle | ko04110 | 50 | 0.0027 | PKMYT1；CDC25B；TGFB3；ANAPC2；LOC101123541；CCND3；CDC26；CDC2；CDKN2C；CDK4；LOC101113385；CCNB1；ATR；CDC26；ENSOARG00000006991；ANAPC4；E2F2；TGFB1；MYC；RB1；CDC14B；E2F3；CCNB3；CCND2；MCM6；TGFB2；CDC27；CHEK1；PRKDC；ATM；MCM4；PTTG1；CDKN1A；E2F5；CDC6；MCM3；CCNA2；BUB1；MAD2L1；CDC7；CDC23；ORC2；CCND1；RBL2；ANAPC11；EP300；CDC14A；MCM5；CDC20；CCNB2 |
| Steroid biosynthesis | ko00100 | 13 | 0.0175 | DHCR7；CEL；DHCR24；NSDHL；SQLE；FAXDC2；EBP；FDFT1；MSMO1；SOAT1；SOAT2；CYP51A1；O.aries_newGene_288 |
| Rap1 signaling pathway | ko04015 | 65 | 0.0337 | KIT；KDR；ID1；PGF；MAP2K3；DOCK4；ADCY9；ACTB；CDH1；FGFR4；PARD6G；PFN2；FGF14；VEGFB；RALGDS；PFN1；NGFR；PDGFA；ANG-2；SKAP1；PIK3CB；HRAS；ADCY8；ENSOARG00000007807；VEGFA；PLCB1；PLCB4；VASP；GNAI2；FGF13；MAGI1；MAP2K2；FLT1；CRK；RRAS；ENSOARG00000013462；BRAF；EGFR；PARD6B；RHOA；MAP2K6；TIAM1；LPAR3；ADORA2A；TEK；ADCY2；ANG-1；SCF；IGF1；RAPGEF6 ；LOC101106246；APBB1IP；GNAS；SIPA1；KRIT1；PFN4；GNAO1；EFNA5；THBS1；NGF；ENSOARG00000020186；ENSOARG00000000431；O.aries_newGene_18622；O.aries_newGene_26746；O.aries_newGene_27167 |
| Proteoglycans in cancer | ko05205 | 64 | 0.0466 | CAV2；KDR；CAV2；HPSE；ACTB；FLNC；IGF2；LOC101120622；SMO；SDC2；MSN；FLNA；ENSOARG00000005717；PIK3CB；HRAS；TGFB1；ENSOARG00000007807；MYC；WNT4；HSPG2；VEGFA；PTCH；FZD2；PRKX；TWIST1；ERBB3；WNT9B；MAP2K2；CD63；TGFB2；ERBB2；GPC3；WNT5B；RRAS；ENSOARG00000013462；BRAF；CDKN1A；EGFR；RHOA；TIAM1；RPS6；WNT5A；DCN；IGF1；ITGA5；LOC101106246；FZD1；ENSOARG00000016991；SDC1；CCND1；MMP2；CBLB；CD44；ENSOARG00000019064；ERBB4；FN1；WNT2B；WNT6；THBS1；O.aries_newGene_15710；O.aries_newGene_18676；O.aries_newGene_27194；O.aries_newGene_27196；O.aries_newGene_27197；O.aries_newGene_4629； |
| Hippo signaling pathway | ko04390 | 50 | 0.0549 | LLGL1；ID1；TGFB3；LATS1；CCND3；ACTB；PPP2R2B；CDH1；LOC101120622；PARD6G；WTIP；BIRC5；TGFB1；MYC；LEF-1；WNT4；FZD2；LLGL2；CCND2；WNT9B；TEAD4；TGFB2；APC2；TEAD3；GDF5；WNT5B；SNAI2；PARD6B；AMH；WWC1；ID2；LOC101115302；WNT5A；DLG3；APC；FZD1；BMP6；AMOT；CCND1；BMP7；LOC101109519；ENSOARG00000019064；WNT2B；WNT6；ENSOARG00000020129；CTNNA2 ；TCF7L1；O.aries_newGene_15310；O.aries_newGene_15710；O.aries_newGene_18676 |

**Supplementary Table S8** DEGs involed in significantly pathway in M6 vs M3 analysis group

| Pathway | ko_id | Number of gene | *P*-value | Gene name |
| --- | --- | --- | --- | --- |
| Focal adhesion | ko04510 | 99 | 0.0049 | LAMA1；RELN；ITGA9；FLT4；DIAPH1；ITGA6；CAV1；COL1A2；MYLK2；MAPK9；PPP1R12C；RAC1；COL5A1；PIK3R3；PIK3R5；VAV2；IBSP；SHC1；PDGFD；ACTN2MAPK10；CHAD；COL1A1；FLNA；ITGA3；ACTN4；PIK3R1；MYLPF；COL27A1；COL4A1；PDGFA；COL4A2；PDGFC；BCAR1；LAMB1；PAK1；LAMC3；ENSOARG00000007807；ITGA1；VEGFA；SHC2；VWF；LAMA4；PAK7；C-JUN；IGF1R；VASP；CCND2；COMP；ERBB2；FIGF；COL6A1；COL6A2；DOCK1；ENSOARG00000013462；LAMA5；BRAF；LAMA2；EGFR；COL5A3；LAMB2；MYL10；CAPN2；MAPK1；MYL9；IGF1；COL5A2；COL3A1；CRKL；ITGA4；ILK；PPP1CC；PRKCB；ITGB1；ZYX；COL4A5；COL4A6；ITGA11；PPP1CC；PDGFRA；COL6A3；LAMC1；PARVG；COL2A1；PAK6；MLCK；ITGB5；COL4A4；COL4A3；SOS2；PIK3CA；SHC4 |
| ECM-receptor interaction | ko04512 | 49 | 0.0052 | LAMA1；RELN；ITGA9；ITGA6；COL1A2；ENSOARG00000002032；COL5A1；IBSP；CHAD；COL1A1；ITGA3；COL27A1；COL4A1；COL4A2；AGRN ；LAMB1；LAMC3；HSPG2；ITGA1；VWFLAMA4；SV2B；COMP；COL6A1；COL6A2；LAMA5；LAMA2；DAG1；COL5A3；HMMR；LAMB2；COL5A2；COL3A1；SV2C；ITGA4；ITGB1；COL4A5；COL4A6；ITGA11；CD44；CD47；COL6A3；LAMC1；COL2A1；ITGB5；COL4A4；COL4A3；O.aries_newGene_5379；O.aries_newGene_9829 |
| Glycerophospholipid metabolism | ko00564 | 50 | 0.0332 | PNPLA7；PLA2G5；PEMT；LOC101107006；ETNK2；AGPAT6；ENSOARG00000002725；PLA2G16；AGPAT9；CDS1；GNPAT；AGPAT2；LPCAT3；TAZ；PGS1；ENSOARG00000005510；PHOSPHO1；PLD3；DGKE；DGKHLOC101104872；PPAP2A；GPD2；PLD4；DGKB；PPAP2C；PLA2G2D；PLA2G3；MBOAT1；LOC101116368；DGKA；AGPAT3；PLA2G1B；LYPLA1；GPAT2；MBOAT2；PLA2G6；CHPT1；LPIN1；LPCAT1；PCYT1B；DGKD；CEPT1；PPP1CB；ETNK1；PCYT1A；PLA2G4E；PLA2G4F；PLD1；O.aries_newGene_24588 |
| Cell cycle | ko04110 | 67 | 0.0392 | MAD2L2；PKMYT1；TGFB3；CDC25A；ANAPC2；CREBBP；CCNE1；CDC2；CDKN2C；SMAD4；YWHAH；SKP1；CDK7；CCNB1；CCNE2；ATR；TTK；E2F2；GADD45G；STAG1MYC；TFDP1；BUB3；ANAPC10；CCND2；RAD21；CCNA1；MCM6；CDK2；GADD45A；CDC27；CHEK1；WEE1；FZR1；MCM4；DBF4；PTTG1；STAG2；WEE2；CDC6；CCNA2；BUB1；ENSOARG00000014422；ANAPC5；YWHAQ；TP53；MAD2L1；CDC7；CDC25C；ESPL1；CDKN2D；ORC2；PCNA；RBL1；PLK1；CDC45；RBL2；CDC14A；SMAD3；CUL1；SMC1B；BUB1B；CDC20；CCNB2；CDKN1B；HDAC1；LOC101102473 |
| Choline metabolism in cancer | ko05231 | 49 | 0.0395 | EIF4EBP1；MAPK9；PLCG1；RAC1；PIK3R3；PIK3R5；MTOR；PDGFD；MAPK10；SLC22A3；ENSOARG00000005510；PIK3R1；PDGFA；PDGFC；DGKE；DGKH；PPAP2A；ENSOARG00000007807；DGKB；PPAP2CC-JUN；WASF1；DGKA；RPS6KB2；PIP5K1C；WAS；SLC44A5；PIP5K1B；ENSOARG00000013462；EGFR；LYPLA1；CHPT1；SLC22A4；MAPK1；PCYT1B；SLC44A3；PRKCB；PDGFRA；DGKD；PCYT1A；PLA2G4E；PLA2G4F；SOS2；PIK3CA；PLD1；HIF1A；O.aries_newGene_18622；O.aries_newGene_22661；O.aries_newGene_26746； |

**Supplementary Table S9** The result lists of DEGs containing continus up-regulation and associated with reproduction terms

| Terms | Gene number | Genes |
| --- | --- | --- |
| reproduction | 23 | CLGN；ODF3；ZNF318；DDIAS；PIWIL1；ENSOARG00000011009；MCM8；PSME4；TMF1；HOOK1；CCT6B；TSSK3；ZPBP；DHX36；SLC4A5；PAQR7；BAZ2B；USP42；ZMYND15；SPATA9；ALMS1；KATNAL1；SPATA19 |
| reproductive process | 23 | ALMS1；KATNAL1；SPATA19；ZMYND15；SPATA9；RBM15；CATSPER2；PAQR7；SLC4A5；USP42；HOOK1；TMF1；CCT6B；ZPBP；DHX36；SPINK4；TSSK3；MCM8；ZNF318；DDIAS；PIWIL1；CLGN；ODF3 |
| developmental process | 157 | CCDC37；ACACB；ENSOARG00000012469；KIF21B；KIF27；ENSOARG00000011379； ACBD7；KIAA1524；S100B；HOOK1；ENSOARG00000001915； STK36；AK8；POM121C；DLEC1；SCLT1；SLC4A5；SPATA19；DNAAF2；CCDC157；ABHD16B；COMP；QRICH2；APBA1；ODF3；RALGPS2；GPR133；ENSOARG00000015512；WDR31；USP42；CEP41；FNIP1；GOLGA4；SYT5；SPATA9；LEKR1；ZMYND15；ANKRD55；PNPLA7；DNAH17；SENP2；TMF1；JARID2；CCDC152；KIAA1683；GOLGA1；PAQR7；KLHL7；MTMR7；PPP1R3E；PASD1；CEP63；NVL；RYR3；BSN；CCDC176；BRPF3；CLIP4；COQ7；CCDC146；BMP2K；PPP6R2；MDM1；WDR38；CCDC18；RIMS2；TSKS；PLEKHG4；LRRC71；ENSOARG00000013362；ZBTB26；ARHGAP26；OSBPL1A；ZDHHC13；PTPN7；PSME4；LRRC29；WDR87；SYCP1；CCDC148；RBM15；GPLD1；ANAPC2；LMNTD2；CCDC62；KBTBD8；C1orf167；C16orf96；KIF5C；WDR62；GNAO1；TSNAXIP1；CNTRL；PIWIL1；ENSOARG00000005999；CLIP1；NEK1；CCPG1；MAP3K13；RUFY3；LCA5；TSSK3；RNF6；TIAM1；CFAP43；SHC4；LOC101102519；USP32；ADAMTS6；LRRC9；KLHL28；MYBL1；CEP164；CEP290；B3GNT4；DDIAS；ZNF318；CTNNA2 ；FAM214B；CDC27；FAM184A；SPINK4；RPGRIP1；ZPBP；FAM161A；DRC1；PKDREJ；WDR60；CATSPER2；LRRIQ1；MLF1；ATP8B3；TTBK2；LRRC57；ATR |
| growth | 2 | APBA1；ENTPD5 |

**Supplementary Table S10** The result lists of DEGs containing continus down-regulation and associated with reproduction terms

| Terms | Gene number | genes |
| --- | --- | --- |
| reproduction | 20 | EVL；RPL10；MMP23B；NANOS2；ACVRL1；PPP1R1B；INHBA；ENSOARG00000003743；NR2F1；RBP4；ENSOARG00000013849；VEGFA；SFRP1；GDF10；RFXANK；CRTAP；ANKRD2；THRA；CCND2；LHR |
| reproductive process | 46 | RPL10；CNTFR；WNT5B；WNT2B；LHX9；EVL；NR2F1；HSP90AB1；ACVRL1；MMP2；PPP1R1B；INSL3；NANOS2；CD44；ALPL；ENSOARG00000013849；GDF5；GLI3；RBP4；TRIP6；IGF2；THRA；INHA；FRZB；NTRK2；NRP1；AMH；WFIKKN2；HSD17B10；FZD2；TWIST1；ENSOARG00000003743；INHBA；FZD1；NRP2；OSR2；VEGFA；LMO4；LHR；VASP；AEBP1；TCF21；CRTAP；IGF1；GDF10；SFRP1 |
| developmental process | 252 | PLD4；FGD1；GPR124；EPHB2；CHGA；DTNB；COLEC12；CD79A；ENSOARG00000009025；ENG；EFS；ACTN4；MYC；HEYL；INHA；CCND2；WFIKKN2；HRC；ALPL；NME4；SEPT6；AIP；OGFR；FSTL3；EFEMP2；GDF5；LRP3；TMEM204；CFL1；TBX2；BACE2；GYPC；FAM212A；PLVAP；AEBP1；KRT86；INPP5J；FKBP10；FHL3；TIMP3；LOC101119195；ENSOARG00000003349；SMO；NES；PSMB8；COL3A1；PLOD1；PPP1R13L；LOC101115302；TRIP6；ENSOARG00000014823；COL5A1；CD44；RAMP2；ENSOARG00000013849；MEIS2；CTDSP1；TUBB；RAB34；TCOF1；C2orf40；BARX1；EVL；CISH；ANKRD2；OLFML3；NID2；GDF10；PTMA；COL9A2；HLA-DRA；LMO4；OSR2；ERBB2；IGLON5；EPHB4；NDRG2；IGF2；TPBG；C-JUN；SYT1；NRP1；AMH；TGFB1I1；BATF3；HOXC8；CAPN1；BAIAP2L2；FLNA；ENSOARG00000008656；HOXC4；PTPRCAP；IGFBP4；CHRNB4；NANOS2；LDLR；PPAPDC3；MXD3；LRFN3；TCN2；TCF21；TPM2；CCDC102A；PKNOX2；CASK；EGR1；GNB2；GAL-1；EFNA5；SLC2A1；FZD1；WNT6；IGFBP7；EBP；CKM；APOE；O.aries_newGene_437；MYO1C；RBP4；ACVRL1；LOC101104530；ID3；MFAP2；TMEM176B；HSPG2；HS3ST3A1；O.aries_newGene_9008；ID2；HOXB6；PDZD11；WNT2B；RARRES2；TMEM119；OLFM2；ACP5；PHB；FAM20C；COL5A2；HSPB6；SNAI2；REM1；CD74；TSKU；MDK；COL6A1；TNKS1BP1；LRRN2；MEIS3；CLEC3B；NRK；ZNF385A；GNB2L1；APCDD1；VIM；PPP1R1B；INSL3；SDC3；PDPN；WNT5B；IGFBP-6；VASP；ANGPTL2；LHR；CRYBB1；SHB ；SFRP1；HOXC5；VEGFA；HPCAL1；MMP14；BIN1；INHBA；IGFBP5；LPCAT1；MFAP4；B4GALNT4；HOXC9；PDGFA；DLK1；TP53I13；NRG1；HES1；NTRK2；VSNL1；ADAM12；MOSPD3；FKBP9；COL6A2；EGFL7；EMILIN1；NR2F1；ENSOARG00000016991；MYL9；ENSOARG00000007807；LRRN3；MAP2K7；LHX9；SDC2；ANPEP；SEPT11；IGF1；C1QA；COL4A1；NGF；DES；NRP2；MPND；SEPT8；EXOC3L2；SH3KBP1；FZD2；CCL5；EPHB3；NLGN4X；THRA；NFATC4；GPC3；PDE7B；SYT17；GLI3；PARVG；LTBR；APOA1；BGN；HSP90AB1；COL1A1；LEPREL2；TMEM184B；HOXD9；IRS4；DUSP9；UNC45B；CHAD；MXRA5；ENSOARG00000003743；PDIA2；MEIS1；C1QTNF2；TWIST1；NGEF；EVC；CLIP2；ASMTL；FRZB；LTBP1；KIF1C；WTIP；LTBP4；COL6A3；PTN；ITM2C；CSK |
| growth | 12 | EVC；FZD1；IGF1；IGF2；NR2F1；SFRP1；SMO；GDF10；VEGFA；PPP1R13L；GLI3；GDF5 |
| hormone secretion | 1 | INHBA |

The following supplementary tables could be seen in the files presented as the excel format.

**Supplementary** **Table S6** GO annotation for DEGs from three analysis group
