# Supplementary material for: Identification of Key Genes Related to Postnatal Testicular Development Based on Transcriptomic Data of Testis in Hu Sheep
Source: Front Genet. 2022 Jan 25;12:773695. doi: 10.3389/fgene.2021.773695 (PMC8822165; doi:10.3389/fgene.2021.773695)
Supplement: Supplementary file 3 [file DataSheet1.ZIP › peer review only/Raw Data Upload.docx]

**Raw Data Upload**

All 11 high-throughput RNA sequencing data analyzed in this study are deposited in Sequence Read Archive (SRA) database in NCBI under accession number PRJNA763055 (https://www.ncbi.nlm.nih.gov/sra/PRJNA763055).

My SRA records will be accessible with the following link after the indicated release date:

<https://www.ncbi.nlm.nih.gov/sra/PRJNA763055>

The details are presented in the BioSampleObjects.xlsx .
